# Oocytes orchestrate protein prenylation for mitochondrial function through selective inactivation of cholesterol biosynthesis in murine species

**DOI:** 10.1016/j.jbc.2023.105183

**Published:** 2023-08-21

**Authors:** Yongjuan Sang, Qiwen Yang, Yueshuai Guo, Xiaofei Liu, Di Shen, Chen Jiang, Xinying Wang, Kang Li, Haiquan Wang, Chaofan Yang, Lijun Ding, Haixiang Sun, Xuejiang Guo, Chaojun Li

**Affiliations:** 1 Modern Animal Research Center of Medical School, Nanjing University, Nanjing, China; 2 State Key Laboratory of Reproductive Medicine and Offspring Health, Nanjing Medical University, Nanjing, China; 3 Center for Reproductive Medicine and Obstetrics and Gynecology, Nanjing Drum Tower Hospital, Nanjing University Medical School, Nanjing, China

**Keywords:** oocyte quality, female infertility, mitochondria, offspring, prenylation

## Abstract

Emerging research and clinical evidence suggest that the metabolic activity of oocytes may play a pivotal role in reproductive anomalies. However, the intrinsic mechanisms governing oocyte development regulated by metabolic enzymes remain largely unknown. Our investigation demonstrates that geranylgeranyl diphosphate synthase1 (Ggps1), the crucial enzyme in the mevalonate pathway responsible for synthesizing isoprenoid metabolite geranylgeranyl pyrophosphate from farnesyl pyrophosphate, is essential for oocyte maturation in mice. Our findings reveal that the deletion of *Ggps1* that prevents protein prenylation in fully grown oocytes leads to subfertility and offspring metabolic defects without affecting follicle development. Oocytes that lack *Ggps1* exhibit disrupted mitochondrial homeostasis and the mitochondrial defects arising from oocytes are inherited by the fetal offspring. Mechanistically, the excessive farnesylation of mitochondrial ribosome protein, Dap3, and decreased levels of small G proteins mediate the mitochondrial dysfunction induced by *Ggps1* deficiency. Additionally, a significant reduction in Ggps1 levels in oocytes is accompanied by offspring defects when females are exposed to a high-cholesterol diet. Collectively, this study establishes that mevalonate pathway–protein prenylation is vital for mitochondrial function in oocyte maturation and provides evidence that the disrupted protein prenylation resulting from an imbalance between farnesyl pyrophosphate and geranylgeranyl pyrophosphate is the major mechanism underlying impairment of oocyte quality induced by high cholesterol.

The mevalonate (MVA) pathway is a core metabolic pathway for multiple cellular processes including producing cholesterols and isoprenoid metabolites that are essential for a variety of biological processes (1). In cells, MVA-derived cholesterol is an important component of most cellular membranes and could also serve as the precursor of steroid hormones (2). Isoprenoid metabolites like geranylgeranyl pyrophosphate (GGPP) and farnesyl pyrophosphate (FPP) contain hydrophobic chains that are essential for the prenylation of proteins, tethering proteins to cell membranes, and enabling proper protein localization and function (3). However, how to balance the two different bypass pathways in the same cell type or tissue remains to be elucidated.

Ovaries are found composed of various cells with metabolic preferences. In ovaries, oocytes are less able to synthesize cholesterol while surrounding cumulus cells provide the products of the cholesterol biosynthetic pathway (4). Loss of cholesterol synthesis in cumulus cells affects female fertility, while deficiency of protein prenylation is dispensable for cumulus cells (5, 6). *In situ* hybridization reveals that robust levels of *Mvk*, *Fdps*, *Sqle*, *Cyp51*, and *Sc4mol* transcripts and *Pmvk* and *Ebp* transcripts were detected in cumulus cells and the periantral granulosa cells, but not in oocytes (4). This led us to hypothesize that in addition to intrinsic metabolic cooperativity between mouse cumulus cells and oocytes, oocytes themselves may have a rationale for shutting down the MVA–*de novo* cholesterol synthesis pathway.

Geranylgeranyl diphosphate synthase1 (Ggps1) is an important branching enzyme that catalyzes the synthesis of GGPP from FPP, both being substrates for protein prenylation. Small GTP proteins are a subset of prenylated proteins between an inactive GDP-bound form and an active GTP-bound form, including Ras and Rho (7, 8). Covalent attachment of geranylgeranyl or farnesyl isoprenoid chain to these proteins promotes their membrane location and exchange for GTP-bound form. It is reported that a lot of small GTPases, such as the Rab family play roles in oocytes maturation (9, 10, 11), indicating the importance of protein prenylation for fully grown oocytes. However, it remains unclear how protein prenylation is regulated to ensure the production of high-quality eggs.

The present study aims to elucidate the specific functions of Ggps1 in growing oocytes, particularly with regards to oocyte maturation, developmental competence acquisition, and response to environmental challenges in mammals. Using a *Ggps1-knockout* mouse model, we showed that *Ggps1*-deleted females exhibited poor oocyte quality and reduced fertility due to impaired mitochondrial function in ovulated oocytes. The dysfunction of mitochondrial activity caused by *Ggps1* deficiency is attributable to both the excess farnesylation of mitochondrial ribosome protein, Dap3, and the reduced levels of small G proteins. Furthermore, we observed substantial reductions of Ggps1 in mature oocytes obtained from mice fed a high-fat and high-cholesterol diet (HF-HCD). Thus, maintaining appropriate protein prenylation in oocytes and cholesterol biosynthesis in granulosa cells benefits female fertility and optimal offspring growth.

## Results

### Small GTP proteins undergoing prenylation showed dynamic expression patterns among oocytes maturation

**Figure 1:**
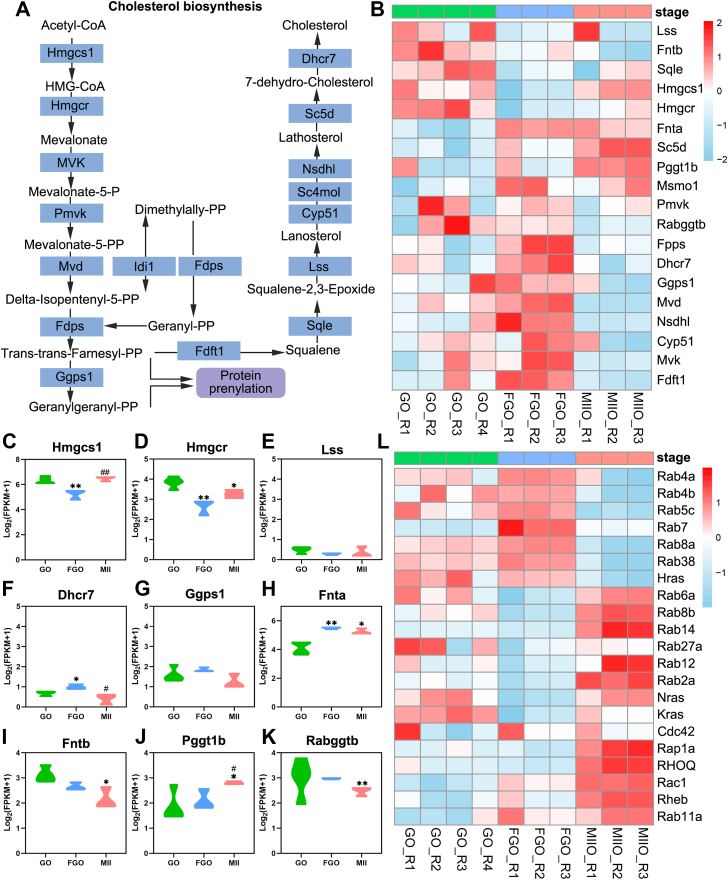
**Small GTP proteins undergoing prenylation showed dynamic expression patterns among oocytes maturation.***A*, schematic diagram of cholesterol *de novo* synthesis pathway. The *blue rectangles* represent the metabolic enzymes. Endogenous cholesterol is synthesized from acetyl-CoA, derived from glucose, glutamine, and/or acetate metabolism. MVA pathway provides substrates to produce sterols and isoprenoid metabolites that are essential for a variety of biological processes. *B*, heatmap showing the dynamics of genes of cholesterol *de novo* synthesis pathway and protein prenylation from GO to MII oocytes in 6-week-old mice. GO: growing oocytes (n = 4). FGO: fully growing oocytes (n = 3). MII: MII-arrested oocytes (n = 3). Three stages are displayed in *green*, *blue,* and *pink*. *C*–*K*, violin plots showing the expression levels (Log2[FPKM+1]) of genes in cholesterol *de novo* synthesis pathway and protein prenylation in mouse oocytes at different stages (n = 3–4, ∗*p* < 0.05, ∗∗*p* < 0.01, ^#^*p* < 0.05, ^##^*p* < 0.01). *L*, heatmap showing the dynamics of genes coding small GTP proteins from GO to MII oocytes in mice (n = 3–4). ∗*p* < 0.05, ∗∗*p* < 0.01, compared with the GO by Student’s *t* test. ^#^*p* < 0.05, ^##^*p* < 0.01, compared with the FGO by Student’s *t* test. The data represent the mean ± SEM. See also Fig. S1. MVA, mevalonate.

MVA pathway provides substrates for cholesterol biosynthesis (Fig. 1*A*). Our previous work demonstrates that MVA pathway is not totally inhibited as cholesterol biosynthesis because oocyte-specific *Ggps1* deletion caused ovarian defects (12). We tried to use single-cell transcriptomics of mouse oocytes to reveal MVA-related gene expression patterns (Fig. S1*A*). Some upstream genes of MVA pathway has relatively high expression during oocyte maturation, such as *Hmgcs1* and *Hmgcr* (Fig. 1, *B*–*D*). Consistent with previous findings, most cholesterol biosynthesis-related genes showed low expression and even had nearly undetected levels in oocytes, such as *Lss* and *Dhcr7* (Fig. 1, *B*, *E*, and F).

Interestingly, we noticed that some prenyltransferases on the bypass pathway of MVA pathway showed opposite expression patterns from GO to MII oocytes. Fnta functioned as the common subunits of FTase and GGTase type I, whose corresponding partners, *Fntb* and *Pggt1b* showed opposite changes, compared with GO oocytes, while *Rabggtb* showed decreases and the branching enzyme, *Ggps1* showed minor change (Figs. 1, *B*, *G*, *H*, *I*, *J*, and *K*). Especially, among oocyte maturation, we found that Rab family proteins, which undergo geranylgeranylation showed dynamic expression patterns in the transition from growing oocytes to MII-arrested oocytes (Figs. 1*L* and S1, *B*–*E*). Being aware of the characteristic of active small GTP proteins relying on membrane location, we were curious about that how can these small GTP proteins achieve functional activation. We hypothesized that metabolites of MVA pathways might support the bypass pathway for protein prenylation rather than cholesterol *de novo* biosynthesis. Combined with previous findings, we provided the idea that Ggps1, a branching enzyme located at the MVA pathway, might play a crucial effect in the process.

### *Ggps1* deficiency in growing oocytes impairs female fertility and embryonic development competency

**Figure 2:**
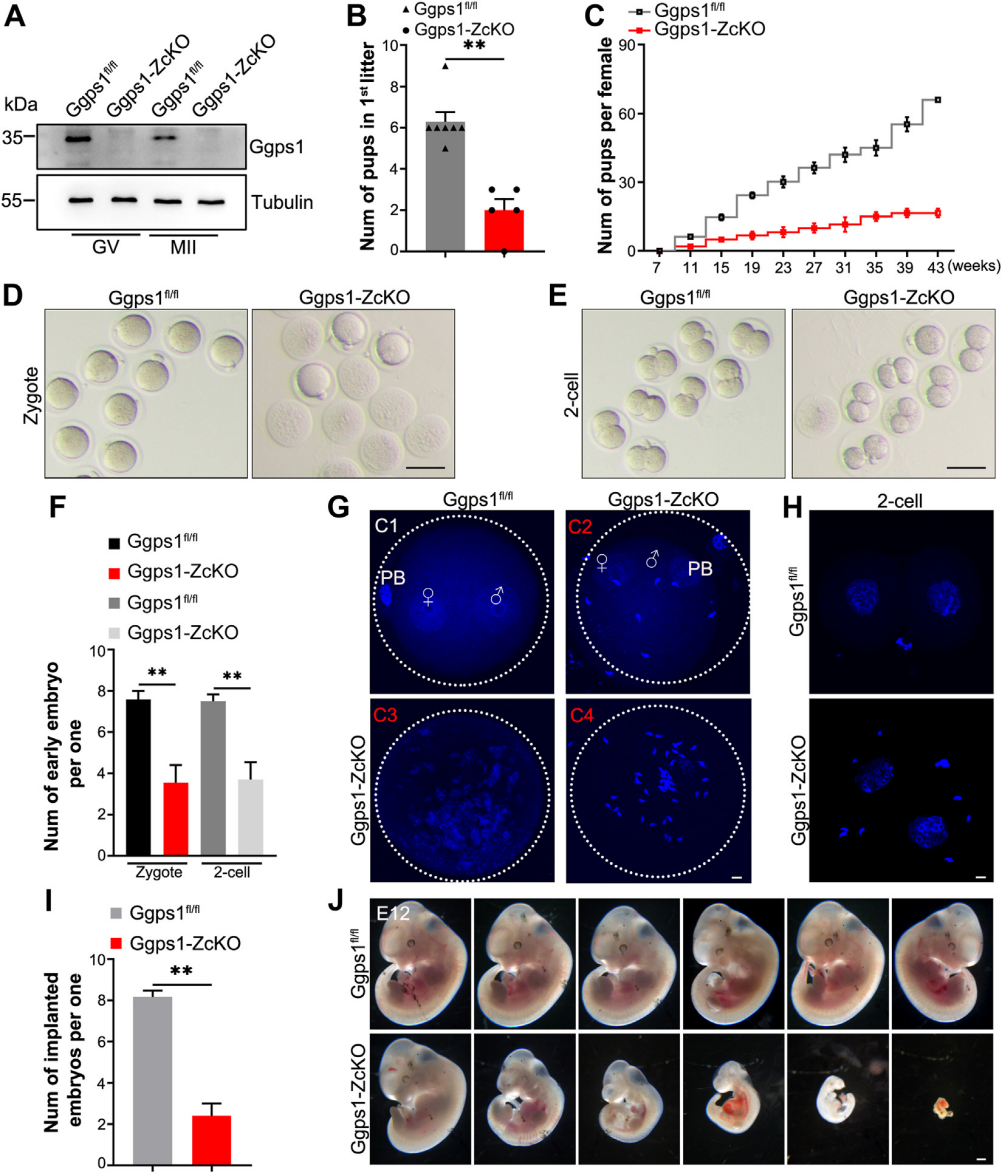
***Ggps1* deficiency in growing oocytes impairs female fertility.***A*, representative Western blot analysis of Ggps1 in GV and MII oocytes obtained from 3-week-old *Ggps1*^*fl/fl*^ and *Ggps1-ZcKO* female mice (n = 3–5, 100∼150 oocytes per lane). Tubulin served as an internal control. *B* and *C*, number of pups born in the first litter and cumulative fertility test of *Ggps1*^*fl/fl*^ and *Ggps1-ZcKO* females (n = 5–7, ∗∗*p* < 0.01). One C57BL/6J male mouse was mated with two female mice. The litter size of each female mouse was continuously recorded over a period of approximately 5 months. *D* and *E*, representative images showing zygotes and 2-cell early embryos from *Ggps1*^*fl/fl*^ and *Ggps1-ZcKO* female mice. Pregnant mice were euthanatized at 0.5 dpc and 1.5 dpc for harvesting zygotes and 2-cell early embryos, respectively (6–8 weeks, pregnant mice, n = 9–12, 30–60 embryos/group). Scale bar, 100 μm. *F*, quantification of zygotes and 2-cell early embryos from *Ggps1*^*fl/fl*^ and *Ggps1-ZcKO* female mice (6–8 weeks, pregnant mice, n = 9–12, ∗∗*p* < 0.01). *G*, confocal images of 2PN zygotes obtained from *Ggps1*^*fl/fl*^ and *Ggps1-ZcKO* oocytes 6 to 8 h after sperm–egg mixing. Fertilized *Ggps1*^*fl/fl*^ oocytes exhibited a normal 2PN stage (C1), whereas much fewer fertilized *Ggps1-ZcKO* oocytes exhibited a normal 2PN stage (C2), and most of them showed defects such as the absence of a female and male pronucleus (C3) or polyspermy (C4). Chromosomes are stained *blue* with DAPI. Scale bar, 10 μm. PB = polar body, PN = pronucleus (6–8 weeks, pregnant mice, n = 6, 20–50 embryos/group). *H*, DAPI staining of 2-cell embryos from *Ggps1*^*fl/fl*^ and *Ggps1-ZcKO* mice (6–8 weeks, pregnant mice, n = 6, 20–50 embryos/group). Scale bar, 10 μm. *I*, assessment of the implantation potential of blastocysts derived from *Ggps1*^*fl/fl*^ and *Ggps1-ZcKO* oocytes. Evans blue staining showing the implantation sites in the uteri of pregnant mice at 4.5 dpc (6–8 weeks, pregnant mice, n = 5–6, ∗∗*p* < 0.01). *J*, representative images showing embryonic developmental defects at E12 in *Ggps1-ZcKO* mice (6–8 weeks, pregnant mice, n = 6–8, 30–60 embryos/group). The embryos were obtained from pregnant mice at 12 dpc. Scale bar, 500 μm. ∗∗*p* < 0.01, compared with the WT or control by Student’s *t* test. The data represent the mean ± SEM. See also Fig. S2. Ggps1, geranylgeranyl diphosphate synthase 1.

We constructed *Ggps1*-knockout mice with Zp3 Cre recombinase (13); the resulting mice were referred to as *Ggps1-ZcKO* mice. Both immunohistochemistry (IHC) and Western blot analyses showed that Ggps1 levels were specifically reduced in growing oocytes and all subsequent oocyte stages in *Ggps1-ZcKO* ovaries (Figs. 2*A* and S2*A*). *Ggps1-ZcKO* females were subfertile with a decreased pup number (Figs. 2, *B* and *C* and S2*B*). During 5 to 6 months of breeding, *Ggps1-ZcKO* females produced fewer pups than WT females (Fig. 2*C*). We then further observed the effect of *Ggps1* deficiency in growing oocytes on embryonic development. We collected early embryos from the oviducts and uterus, and gross observation showed that although there were no morphological differences after *Ggps1* deletion, the numbers of fertilized zygotes and 2-cell embryos declined (Fig. 2, *D*–*F*). DAPI staining showed defects, such as the absence of a female and male pronucleus or polyspermy (Fig. 2, *G* and *H*). Examination of implanted embryos indicated that embryo implantation was largely decreased at embryonic day (E) 4.5 (Fig. 2*I*) and that embryonic development was significantly blocked at different stages at E12 (Fig. 2*J*).

Although the development of surviving ZcKO-F1 embryos derived from Ggps1-ZcKO females appeared normal, their postnatal body weights were observed to be higher, possibly due to the increased space and nutrient supply in the uterus resulting from smaller fetal size, since embryo transfer of *Ggps1-ZcKO* embryos eliminated this dissimilarity (Fig. S2, *C* and *D*). Although no difference was observed in body weight after weaning, the *ZcKO-F1* offspring showed a metabolic disorder phenotype during adulthood (Fig. S2, *E*–*H*). The glucose tolerance test (GTT) and insulin tolerance test (ITT) indicated that the responses of 3-month-old adult mice were heterogeneous (Fig. S2, *E* and *F*). Nine-month-old *ZcKO-F1* mice showed improved glucose tolerance and a decreased insulin sensitivity phenotype (Fig. S2, *G* and *H*). Thus, *Ggps1* deficiency in growing oocytes results in defective fertility and damages metabolic homeostasis in offspring.

### *Ggps1* deficiency in growing oocytes results in oocyte meiosis defects

We next examined follicle development in *Ggps1-ZcKO* mice. There were no ovarian weight differences or morphological changes in *Ggps1-ZcKO* females (Fig. S3, *A* and *B*). Follicle development quantification indicated that *Ggps1* deletion in growing oocytes did not impair follicle development (Fig. S3*C*). The ovulation rates were also comparable with an average of 40 oocytes of *Ggps1-ZcKO* females after superovulation, similar to the number for WT females (Fig. S3*D*).

Oocyte meiotic progression was then analyzed to assess possible mechanisms for the diminishment of egg quality in *ZcKO* females. Although *Ggps1-ZcKO* oocytes produced nearly the same quantity of MII oocytes as WT oocytes, some *Ggps1-ZcKO* oocytes exhibited meiosis abnormalities that included chromosome misalignment at metaphase, clumped chromosomes, and spindle apparatus defects (Fig. S4*A*). However, the first polar body (PB1) extrusion rate in natural ovulation only slightly decreased (Fig. S4*B*). Furthermore, these defects were recapitulated when *Ggps1-ZcKO* oocytes underwent *in vitro* maturation, although GVBD progression and the emission extrusion rate of the first polar body were not severely affected (Fig. S4, *C* and *D*). Approximately 17% of *Ggps1-ZcKO* oocytes at the GV stage even extruded oversized PB1s during *in vitro* maturation (Fig. S4, *E* and *F*), indicating that oocyte cytokinesis was partially disrupted in *Ggps1-ZcKO* oocytes. Moreover, *Ggps1-ZcKO* mice showed more abnormal and more Annexin V positive oocytes (Fig. S4, *G* and *H*). Our results suggest that *Ggps1* deletion affects the meiosis progression of mouse oocytes. Nevertheless, given the significant reduction in female fertility, we postulate that the partial failure of meiotic progression to MII is not the primary cause of the diminished quality of *ZcKO* oocytes.

### Mitochondrial defects in *Ggps1*-deficient oocytes were responsible for poor oocyte quality and inherited by offspring

In mammals, it has been observed that oocytes with a higher number of mitochondrial DNA copies have a more favorable fertilization rate compared to those with a lower number of such copies (14). Also, mitochondria also undergoes a significant increase in membrane potential during oocyte maturation (15). Treatment with statins in some *in vitro* cell lines, such as Jurkat and Kc167 cells, has been found to boost the percentage of larger mitochondria, which is prevented by the addition of mevalonate or GGPP (16). The regulation of organelle remodeling during oocyte maturation occurs through small GTPases, which undergo protein prenylation (9, 10, 11). Therefore, we sought to examine whether GGPP depletion influenced oocyte mitochondria.

**Figure 3:**
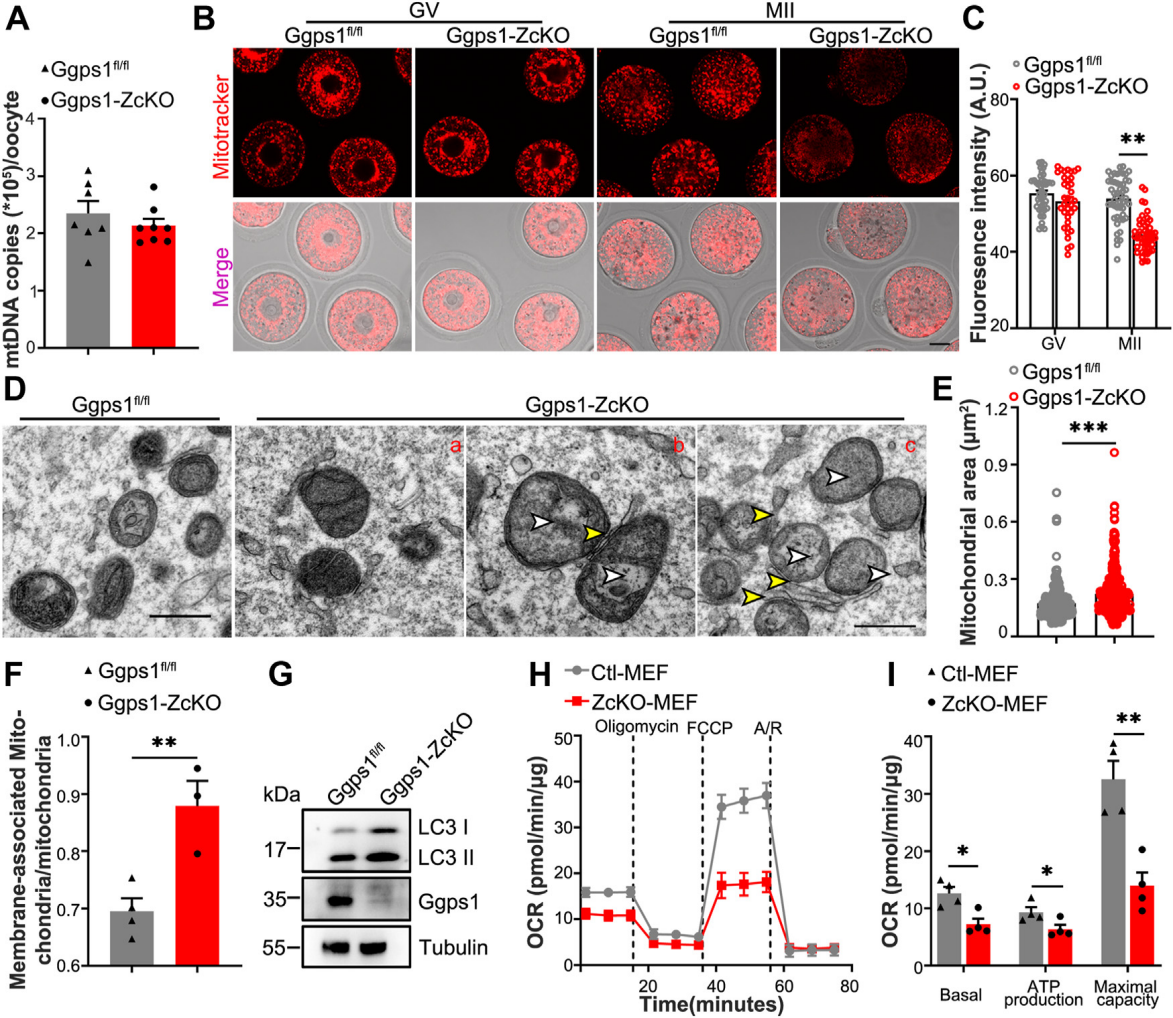
**Mitochondrial defects in *Ggps1*-deficient oocytes.***A*, mtDNA copy number per oocyte in *Ggps1*^*fl/fl*^ and *Ggps1-ZcKO* mice (3 weeks, n = 7–8, 10 oocytes per mouse, ∗*p* < 0.05). *B* and *C*, MitoTracker Red staining and quantification of fluorescence intensity of MitoTracker Red staining in GV and MII oocytes of *Ggps1*^*fl/fl*^ and *Ggps1-ZcKO* mice, respectively. Scale bar, 20 μm. GV oocytes were obtained from mice administrated with eCG 48 h (3 weeks, n = 3–5, 100 oocytes/group). MII oocyte were obtained from mice administrated with eCG 48 h, followed by hCG 13 to 16 h (3 weeks, n = 3–5, 100 oocytes/group, ∗∗*p* < 0.01). *D*, TEM imaging showing the mitochondrial ultrastructure in oocytes of *Ggps1*^*fl/fl*^ and *Ggps1-ZcKO* mice (3 weeks, n = 3–5, ∼100 MII oocytes/group). Scale bar, 500 nm. The *white arrowheads* indicate mitochondrial cristae that are reduced, and the *yellow arrowheads* indicate the contact sites between the membrane and mitochondria*. E*, quantification of mitochondrial areas in MII oocytes of *Ggps1*^*fl/fl*^ and *Ggps1-ZcKO* mice during TEM imaging (3 weeks, n = 3, ∗∗∗*p* < 0.001). *F*, quantification of membrane–mitochondria contacts in MII oocytes of *Ggps1*^*fl/fl*^ and *Ggps1-ZcKO* mice during TEM imaging (3 weeks, n = 3, ∗∗*p* < 0.01). *G*, Western blot analysis of LC3 in oocytes ovulated from *Ggps1*^*fl/fl*^ and *Ggps1-ZcKO* mice (3 weeks, n = 5–7, 150 oocytes per lane). Tubulin served as an internal control. *H* and *I*, OCR analysis of MEFs isolated from embryos at E13.5 from *Ggps1*^*fl/fl*^ and *Ggps1-ZcKO* pregnant mice (n = 4, ∗*p* < 0.05 and ∗∗*p* < 0.01). The assay was performed with an XF Cell Mito Stress Test Kit. ∗*p* < 0.05, ∗∗*p* < 0.01, ∗∗∗*p* < 0.001, compared with the WT or control by Student’s *t* test. The data represent the mean ± SEM. See also Fig. S5. Ggps1, geranylgeranyl diphosphate synthase 1; MEFs, mouse embryonic fibroblasts; OCR, oxygen consumption rate; TEM, transmission electron microscopy.

We found that *Ggps1* deletion in growing oocytes only slightly decreased mitochondrial DNA copy numbers without causing a significant difference (Fig. 3*A*). We then compared the mitochondrial potential in GV and MII oocytes with MitoTracker Red staining. In GV oocytes, MitoTracker Red fluorescence was mainly found in the perinuclear region (Fig. 3*B*), and the average fluorescence intensity was comparable between control and *Ggps1-ZcKO* oocytes (Fig. 3*C*). However, in MII oocytes, MitoTracker Red fluorescence was uniformly distributed in the oocyte cytoplasm, but *Ggps1-ZcKO* oocytes showed decreased average fluorescence intensity (Fig. 3, *B* and *C*), indicating impaired mitochondrial potential. Additionally, transmission electron microscopy examination showed that *Ggps1* deficiency largely impaired mitochondrial structure (Fig. 3*D*). There were more hollow mitochondria with fewer cristae and more enlarged mitochondria in *Ggps1*-deficient MII oocytes, and more mitochondria were associated with other membrane structures in these oocytes (Fig. 3, *D*–*F*). The defects in mitochondria were also indicated by the increased levels of the autophagy marker LC3 in *Ggps1-ZcKO* MII oocytes, which might be associated with increasing defected mitochondria or inhibited lysosomal mitochondrial clearance (Fig. 3*G*). These data show that *Ggps1* deficiency leads to mitochondrial dysfunction during oocyte maturation by mitochondrial activity rather than quantity.

Mitochondrial dysfunction not only is detrimental to oocyte competency but also increases the risks of poor embryogenesis and abnormal offspring (17). To evaluate the mitochondrial activity in embryos derived from *ZcKO* oocytes, we isolated mouse embryonic fibroblasts (MEFs) from E13.5 embryos of each group. Of note, extracellular metabolic flow analysis of the oxygen consumption rates (OCRs) of MEFs showed decreased mitochondrial activity in *ZcKO*-MEFs, including consistent reductions in basal and maximum respiration capacity (Fig. 3, *H* and *I*). Additionally, we found imbalanced expression of subunits of the respiration chain complex in MEFs from *Ggps1-ZcKO* mice (Fig. S5, *A* and *B*). In sum, these data indicate that mitochondrial defects derived from ZcKO oocytes are inherited by the fetal offspring.

### Proteomics reveal metabolic pathway alterations and reduced small GTPases in *Ggps1*-deficient oocytes

**Figure 4:**
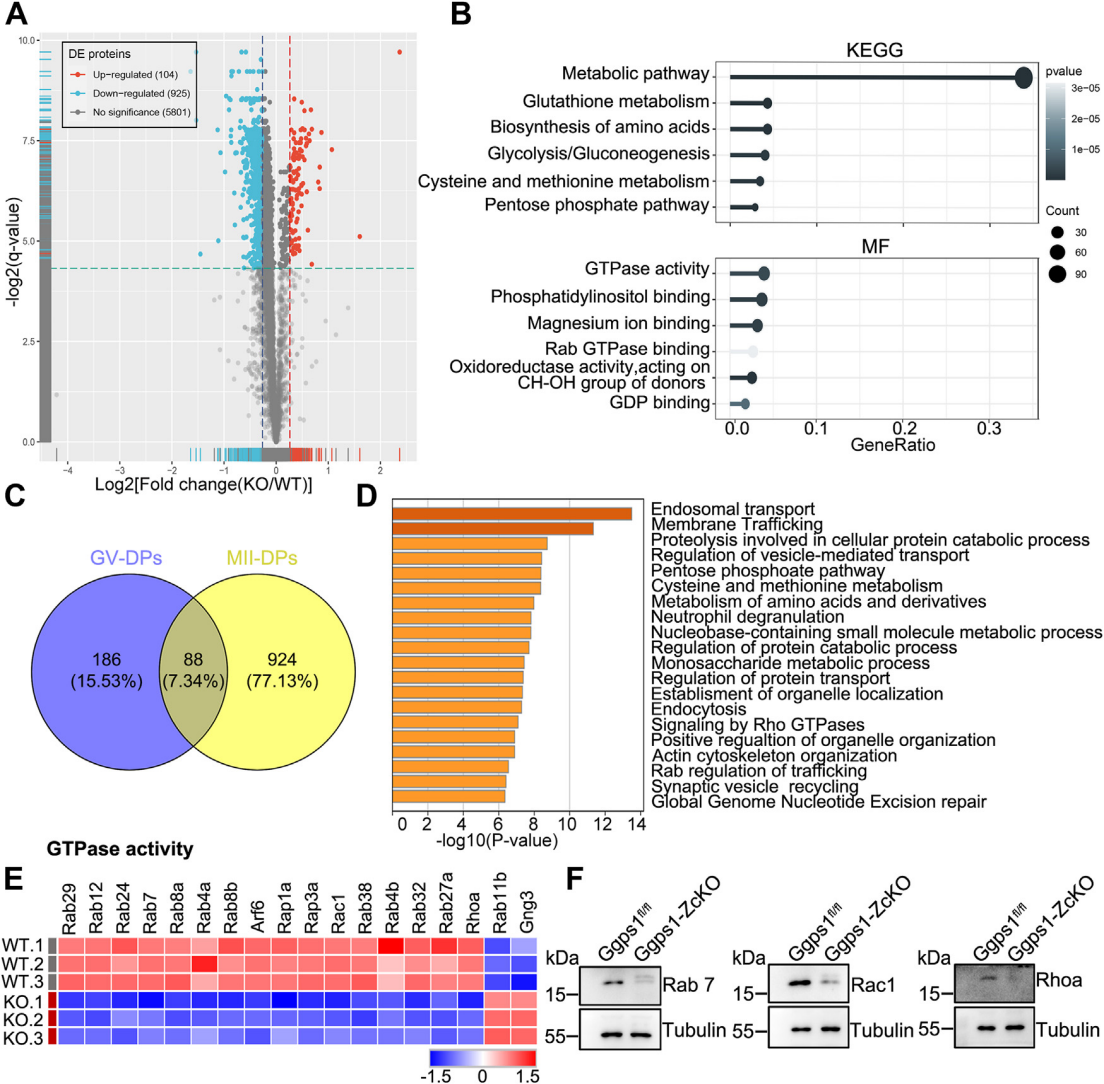
**Proteomics reveal metabolic pathway alterations and reduced small GTPases in *Ggps1-*deficient oocytes.***A*, distribution of significantly changed proteins at various magnitudes of difference in expression levels between *Ggps1*^*fl/fl*^ and *Ggps1-ZcKO* ovulated oocytes detected by LC–MS/MS. The number of changed proteins in each category of fold change is indicated above each bar (3 weeks, n = 60–80, 2000 oocytes per group, fold change ≥ 1.2). *B*, heatmaps illustrating the enriched terms (KEGG terms and MF) identified by LC-MS/MS in ovulated *Ggps1*^*fl/fl*^ and *Ggps1-ZcKO* oocytes. The proteins being analyzed are those with significant changes (∗*p* < 0.05, fold change ≥ 1.2). *C*, Venn diagram illustrating the relationships of the changed proteins identified by LC-MS/MS in *Ggps1*^*fl/fl*^ and *Ggps1-ZcKO* GV and ovulated oocytes. DPs: differential proteins (∗*p* < 0.05, fold change ≥ 1.2). *D*, heatmaps illustrating the enriched terms of proteins that were independent in the *Ggps1-ZcKO–*ovulated oocytes. Heatmap of enriched terms was created by metascape (http://metascape.org/). *E*, heatmaps illustrating differences between *Ggps1*^*fl/fl*^ and *Ggps1-ZcKO* ovulated oocytes in the expression of proteins that were involved in GTPase activity. To perform LC/MS analysis, ovulated oocytes from *Ggps1*^*fl/fl*^ and *Ggps1-ZcKO* were divided into three replicates. *F*, identification of changes in small GTPases in ovulated oocytes by Western blot (n = 5–7, 150 oocytes per group). Tubulin served as an internal control. See also Fig S6 and Table S2. Ggps1, geranylgeranyl diphosphate synthase 1; LC-MS/MS, liquid chromatography-tandem mass spectrometry.

Disrupted mitochondria are responsible for impaired oocyte development competency and failed embryogenesis (18, 19). To unravel the potential molecular mechanisms underlying the defects in mitochondria of *Ggps1-ZcKO* oocytes, we performed a comparative proteomic analysis of GV and MII oocytes from WT and *Ggps1-ZcKO* mice. In liquid chromatography-tandem mass spectrometry (LC–MS/MS) analysis and label-free quantification, only 281 proteins were identified to be significantly changed in the *Ggps1-ZcKO* oocytes as compared with WT at the GV stage, of which 100 proteins were downregulated, and 181 proteins were upregulated, respectively (Fig. S6, *A* and *B*). In MII oocytes, a total of 1029 proteins were identified to be significantly changed in *Ggps1-ZcKO* oocytes compared with WT oocytes, of which 925 proteins were downregulated and 104 proteins were upregulated, respectively (Fig. 4*A*). KEGG analysis in both stages showed that the “metabolic pathways” is the most significant changed processes among the GO terms (Figs. 4*B* and S6*C*). This result supports our findings of defective mitochondria in *Ggps1-ZcKO* mice, as it is well known that mitochondria reside at the centre of a complex network of metabolic pathways including glucose, fatty acids, and amino acids that can be modulated to counterbalance reductions in Oxidative phosphorylation to maintain cellular homeostasis and mitochondrial dysfunction has been found to rewire cell metabolism in multiple tissues (Fig. S6*D*) (20, 21, 22, 23, 24, 25).

Considering the finding that impaired mitochondria occurred until the MII stage rather than the GV stage, we proposed that some specific differentially expressed proteins at MII rather than the GV stage play critical roles during oocyte maturation. Compared with that in GV oocytes, the proteins were changed more profoundly in MII oocytes, with a significant increase in the number of downregulated proteins (Fig. 4*C*). Bioinformatics analysis of independently changed proteins at the MII stage showed that “endosomal transport” and “membrane trafficking” were among the GO terms associated with downregulated proteins (Fig. 4*D*). Of note, bioinformatics analysis revealed that some small GTPases were downregulated consistently (Fig. 4*E*), such as those in the Rab family. Downregulation of representative proteins in the family including Rab7, Rac1, and Rhoa were validated by Western blot (Fig. 4*F*). It has been reported that some small GTPases are involved in endosome pathway-mediated mitochondrial clearance, such as Rab5 and Rab7. Very few papers have reported that inhibition of Ggps1 in the mevalonate pathway leads to reductions of target proteins, although it has usually been found to be related to diminished protein geranylgeranylation in our previous work (26, 27). Collectively, these data imply that a defective small GTPase is a universal lesion in disturbed protein prenylation-induced mitochondrial dysfunction.

### Excessive farnesylation of mitochondrial protein, Dap3 in *Ggps1*-KD cells, is responsible for impaired mitochondrial function

Several papers have reported that disruption of the MVA pathway affects mitochondrial morphology and function (16, 28) in which some proteins undergoing prenylation serve to mitochondrial quality control by affecting autophagy or metabolic enzymes (29). Therefore, we postulated that protein prenylation might be one of the PTMs responsible for mitochondrial functions.

**Figure 5:**
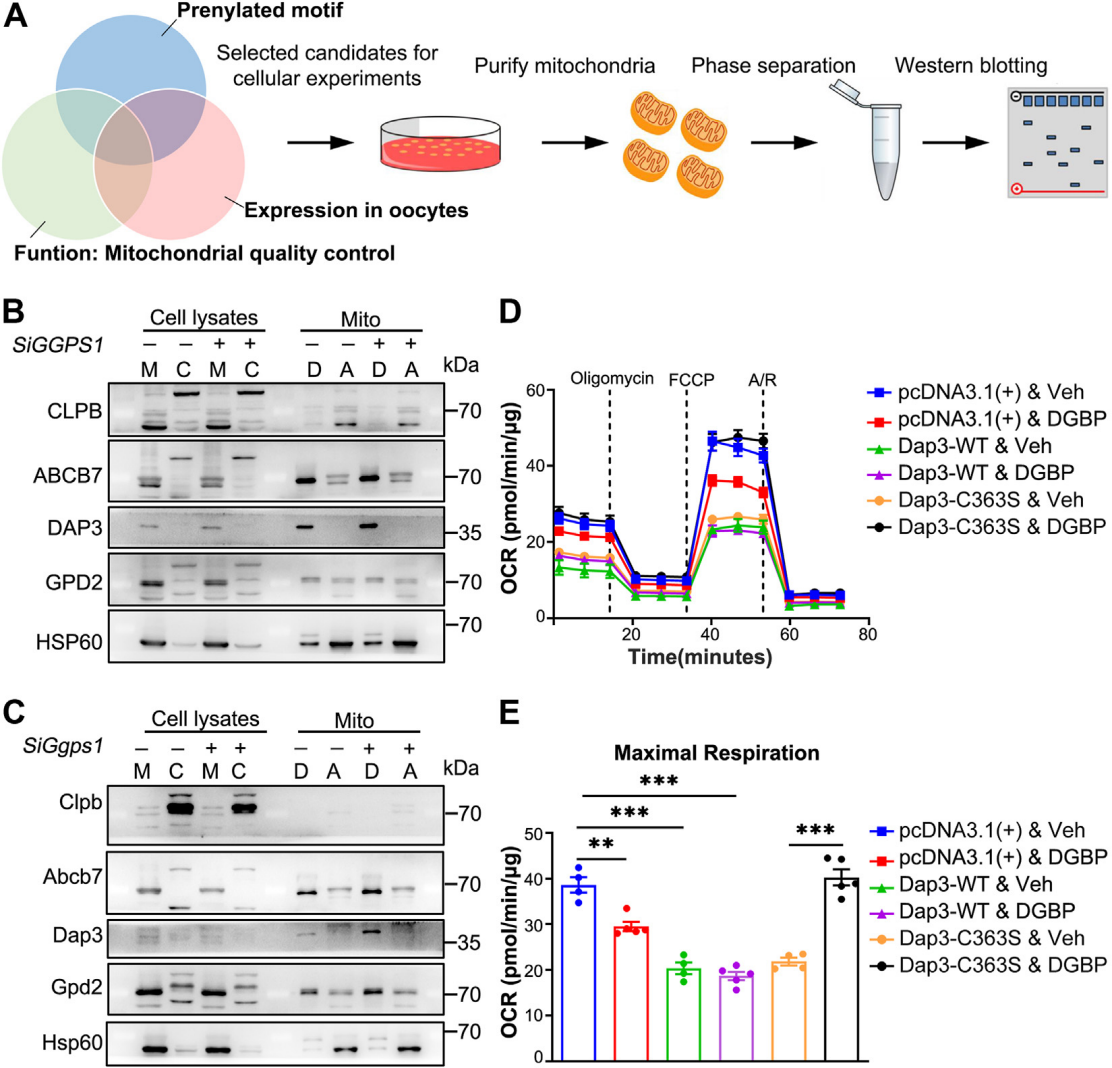
**Excessive farnesylation of Dap3 in *Ggps1*-KD cells is responsible for impaired mitochondrial function.***A*, schematic of the screening of prenylated proteins in mitochondria of *Ggps1*-KD cells *in vitro*. Protein prenylation was assessed by immunoblotting. *B* and *C*, aqueous phase-localized and detergent phase-localized candidate proteins in mitochondrial components of control and *Ggps1*-KD cells were obtained *via* Triton X-114 extraction and analyzed by immunoblotting. Assays were performed in 293T cells (*B*) and Aml12 cells (*C*). Hsp60 served as mitochondrial markers. M: mitochondria, C: cytosol, D: detergent phase, A: aqueous phase. The experiments were repeated three times. *D* and *E*, oxygen consumption rates (OCRs) and maximal respiration in Aml12 cells were detected with an XF Cell Mito Stress Test. We cultured Aml12 cells transfected with vector, Dap3-WT, or Dap3-C363S mutant for 48 h with or without 10 μM DGBP treatment for 24 h. DGBP was used to inhibit Ggps1 activity. (n = 3, ∗∗*p* < 0.01 and ∗∗∗*p* < 0.001). The experiments were repeated three times. ∗∗*p* < 0.01, ∗∗∗*p* < 0.001, compared with the WT or control by Student’s *t* test. The data represent the mean ± SEM. See also Table S3. DGBP, digeranyl bisphosphonate; Ggps1, geranylgeranyl diphosphate synthase1.

We expected to find novel mitochondria-related prenylated proteins in addition to canonical small G proteins. Based on the features of the modified protein motif (30). We screened and validated the potential mitochondria-related prenylated protein *in vitro* according to the work-flowing method (Fig. 5*A*). We found no proteins showing altered subcellular localization in the *Ggps1*-KD group in 293T cells, and the results were similar in mouse cell line, Aml12 (Fig. 5, *B* and *C*). Furthermore, we wanted to determine whether proteins in mitochondria have different localizations between the aqueous and detergent phases. Among the detected proteins, Dap3 showed increased detergent localization in the *Ggps1*-KD group in both two cell lines, although the mitochondrial localization did not change (Fig. 5, *B* and *C*). This finding indicated that Dap3 underwent enhanced protein farnesylation in the *Ggps1*-KD cells, based on the prenylated motif and the ratio of aqueous and detergent localization. Both human and mouse Dap3 contain the CASL amino acid motif in the C terminus as potential modification sites for prenyltransferase. We substituted the cystine for serine. The wildtype and mutants were overexpressed in Aml12 cell lines with or without digeranyl bisphosphonate (DGBP), a powerful Ggps1 inhibitor. Consistent with a previous study, the DGBP reduced mitochondrial respiration ability (Fig. 5, *D* and *E*). Furthermore, overexpression of either wildtype or mutant Dap3 led to impaired mitochondrial respiration ability (Fig. 5, *D* and *E*). However, the DGBP-induced inhibition of OCRs was rescued with mutant Dap3, indicating that Ggps1-regulated mitochondrial respiration depends on farnesylation of Dap3. Thus, these data show that enhanced farnesylation of Dap3 mediates *Ggps1* deficiency-induced impairment of mitochondrial function.

### Hypercholesterolemia disrupts ovarian protein prenylation and results in offspring metabolic dysfunction

**Figure 6:**
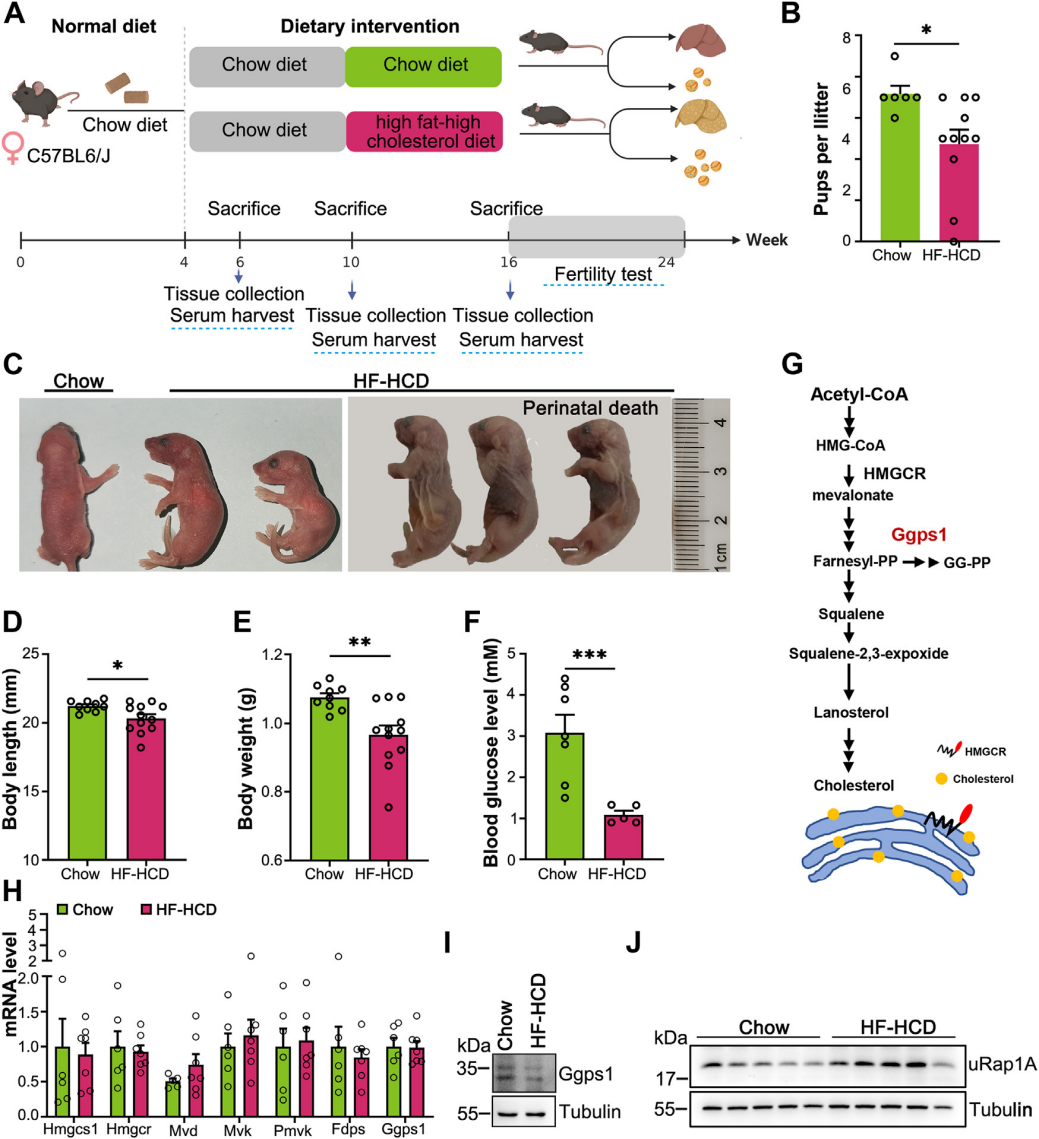
**Hypercholesterolemia disrupts ovarian protein prenylation and results in offspring metabolic dysfunction.***A*, schematic diagram of a dietary regimen of C57BL/6J female mice and schematic representation of the experimental procedures used in the HF-HCD model (n = 5–9/timepoint). *B* and *C*, the fertility of female HF-HCD mice. The average litter size was slightly reduced in HF-HCD mice compared to Chow mice when these mice (HF-HCD for 16 weeks) were mated with wildtype males (females, n = 6–11, ∗*p* < 0.05). *C*, representative images showing fetuses morphology at P1. The female mice were fed with HF-HCD from 6 weeks to 24 weeks. *D*–*F*, newborn mice were assessed for body length (*D*, n = 9–12) and body weight (*E*, n = 9–12) at P1. Blood glucose of newborn mice were tested in P2 (*F*, n = 5–7) before death. ∗*p* < 0.05, ∗∗*p* < 0.01 and ∗∗∗*p* < 0.001. *G*, schematic illustration of the MVA pathway and cholesterol synthesis. *H*, mRNA levels of cholesterol biosynthesis-related genes in the ovaries of mice fed Chow or a HF-HCD for 10 weeks (n = 8, ∗*p* < 0.05). *I*, Western blot analysis of Ggps1 in oocytes of mice fed normal chow or a HF-HCD for 16 weeks (∼120 oocytes from 5–7 mice in each group). Tubulin served as an internal control. *J*, Western blot analysis of prenylation of Rap 1A in the ovaries of mice fed normal chow or a HF-HCD for 10 weeks (n = 5). Tubulin served as an internal control. ∗*p* < 0.05, ∗∗*p* < 0.01, ∗∗∗*p* < 0.001, compared with the WT or control by Student’s *t* test. The data represent the mean ± SEM. See also Fig. S7. HF-HCD, high-fat and high-cholesterol diet; MVA, mevalonate.

Cellular pathways are coordinated to maintain cholesterol homeostasis, particularly in cholesterol-rich environments (31). To establish a high-cholesterol diet mouse model, we began feeding female mice with HF-HCD at 3 weeks. Despite the lack of significant difference in body weights among the female mice, the liver weights and cholesterol levels in the blood and liver were significantly enhanced after HF-HCD exposure (Fig. S7, *A*–*D*). In addition, the female fertility was affected, as evidenced by a decrease in the number of offspring present in the first litter when these females were mated with wildtype chow-fed males (Fig. 6, *A* and *B*). Moreover, exposure to HF-HCD led to postnatal mortality, as approximately two out of three fetuses died on postnatal day 2 (P2) in the HF-HCD group, unlike in the chow-fed group (Fig. 6, *B* and *C*). The offspring exhibited a growth retardation phenotype characterized by a significant decrease in size and body weight at birth (Fig. 6, *C*–*E*). Metabolic analysis revealed that the cause of death for these offspring was severe hypoglycemia, with glucose levels dropping as low as 1 mM prior to their demise (Fig. 6*F*).

Endogenous cholesterol is synthesized from acetyl-CoA in the ER (Fig. 6*G*), a process that is blocked by exogenous cholesterol administration. Although our observations illustrated the significant blockage of endogenous cholesterol biosynthesis in the liver as previously noted (Fig. S7*E*), mouse ovaries were unaffected (Figs. 6*H* and S7*F*). Nevertheless, the protein levels of Ggps1 in oocytes were largely reduced (Figs. 6*I* and S7*G*). Consistently, protein prenylation was also extensively impeded (Figs. 6*J* and S7*H*). We postulated that protein prenylation perturbations might mediate embryonic development and offspring defects that were induced by exposure to HF-HCD.

## Discussion

Inhibition of the MVA pathway has been documented to cause a reduction in the mevalonate pool, resulting in reduced cholesterol biosynthesis, impaired protein prenylation (32, 33). In mammals, cholesterol biosynthesis and protein prenylation are differently required in the cumulus cell–oocytes complex. Cumulus cells are the major origins of cholesterol biosynthesis, while the GGPP-mediated geranylgeranylation is less important because *Ggps1* deletion in cumulus cells has no impact on ovarian function (6, 34, 35, 36), which enhances cellular cholesterol level (unpublish data). Our recent work suggests that downregulated MVA pathway in granulosa cells in aging causes oocyte meiotic defects and aneuploidy (37). Our present study found that the oocyte MVA pathway supports the isoprenoid synthesis rather than *de novo* cholesterol biosynthesis during oocyte maturation. Recently, metabolomic profiling of mouse oocyte maturation shows decreased cholesterol levels in the transition from GV to MII-arrested oocytes (38). Thus, we postulated that enhanced isoprenoid synthesis may be a way to diminish cholesterol accumulation in murine oocytes.

The nematode *Caenorhabditis elegans* (*C. elegans*) and *Drosophila* possess a mevalonate pathway that lacks the branch leading to cholesterol synthesis and thus represents an ideal organism to specifically study the noncholesterol roles of the pathway. Inhibiting HMG-CoA reductase in *C. elegans* using statins or RNAi leads to reduced fertility, slower developmental timing developmental arrest (39, 40). In *Drosophila*, Hmgcr plays a role in the release of the signaling molecule Hedgehog from Hedgehog expressing cells and in the production of an attractant that directs primordial germ cells to migrate to the somatic gonadal precursor cells, and reduced HMGCR activity results in germ cell migration defects (41, 42). In addition, pharmacological HMGCR inhibition alters zebrafish development and germ cell migration, which were completely rescued by prior injection of mevalonate, the product of HMGCR activity, or the prenylation precursors farnesol and geranylgeraniol (42). Together with data from *C. elegans*, *Drosophila,* and zebrafish, our results highlight a conserved role for protein geranylgeranylation in female fertility.

**Figure 7:**
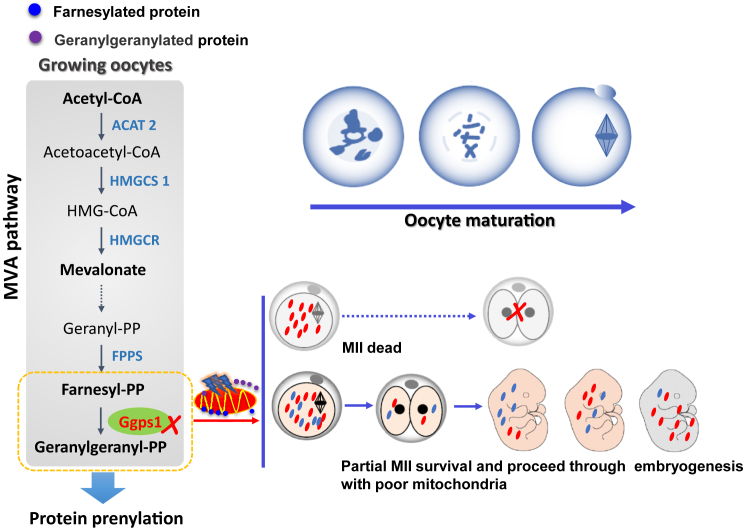
**Protein prenylation in growing oocytes affects offspring outcomes.** The Mevalonate pathway is a core metabolic pathway for cellular processes including producing cholesterol and isoprenoid metabolites. Oocytes MVA pathway supports bypass isoprenoid synthesis rather than cholesterol biosynthesis during oocyte maturation. Ggps1, the branch point enzyme of the mevalonate pathway to synthesize isoprenoid metabolite GGPP from FPP, regulates oocytes maturation by enhancing the geranylgeranylation of protein that is critical for mitochondrial homeostasis and then affects developmental competence. FPP, farnesyl pyrophosphate; GGPP, geranylgeranyl pyrophosphate; MVA, mevalonate.

Research has revealed that *GGPS1* mutations cause muscular dystrophy, hearing loss, and ovarian insufficiency syndrome in patients (43). indicating *GGPS1* has a critical role in humans. In that work, five different biallelic mutations in specific domains of GGPS1 were identified in patients in six families. All postpubertal females had primary ovarian insufficiency, which is consistent with the findings of our genetic work in an animal model (12, 43) revealing that *GGPS1* is essential for early folliculogenesis and oocyte maturation in women fertility. Here, we uncovered oocyte-specific roles of Ggps1 in oocyte maturation and acquisition of developmental competence in mammals in a genetics model. Mechanistically, we have uncovered that the augmented farnesylation of Dap3, combined with lowered levels of small G proteins, contributes to the impairment of mitochondrial function (Fig. 7). Moreover, FPP and GGPP have also been found to directly bind to target noncovalent proteins, which is different from covalent modification (unpublished data).

In the past two decades, mitochondrial number and ultrastructure have been studied in different species, which points to that mitochondria are precisely controlled during oogenesis. In oocytes, the appropriate mitochondrial number and activity ensure oocyte maturation and fertilization outcomes to give a healthy baby. A recent study has implicated that loss of *Lonp1*, a mitochondrial protease compromises oocyte development and results in premature ovarian insufficiency (44). In addition, Drp1-dependent spatiotemporal regulation of mitochondria in oocytes severely affects various types of membrane dynamics and oocyte quality (45). Even though statins are considered to be toxic to mitochondrial function, the mechanism is not clear (16). Our study found that disrupted protein prenylation in growing oocytes led to poor oocyte competency, indicating that protein prenylation functions as a critical factor in the regulation of mitochondrial quality control. Therefore, supplementation of GGPP might be a potential approach for improving clinic oocyte quality.

Cholesterol plays a crucial role in fertility as it is an essential component of steroid hormones, including progesterone and testosterone. Studies in humans have shown that the increased pregnancy failure rate in obese women returns to the normal rate if donor oocytes are used instead of autologous oocytes (46). The HDL receptor scavenger receptor class B type I controls the structure and fate of plasma HDL. Previous report showed that female, but not male, *SR-BI* KO mice exhibit fully penetrant infertility despite their essentially normal ovarian histology due to excess cholesterol deposition in oocytes (5). Reversing the abnormal plasma microenvironment of high plasma HDL-mol% FC in female *Scarb1* mice rescues fertility (47). Moreover, cholesterol depletion in plasma and tissues in a mouse model deficient for the gene encoding 24-dehydrocholesterol reductase (*Dhcr24*) leads to mouse infertility (48). It was reported that hypercholesterolemia in rabbits modifies sperm morphology and function in combination with changes in plasma membrane composition (49). Similarly, dietary cholesterol overload in the *Liver-X-Receptor* knockout mice led to sperm abnormalities, including morphological changes, decreased motility, and capacitation failure, as observed in hypercholesterolemic rabbits (50). The current investigation found that inhibition of protein prenylation by *Ggps1* depletion in mature oocytes impaired oocyte quality, revealing that reductions of Ggps1 in mature oocytes may play a role in impaired fertility in mice fed a HF-HCD diet. Thus, cholesterol imbalance may have negative impacts on reproductive function.

The aforementioned findings reinforce the unfavorable consequences of excess cholesterol on fertility. Our research has highlighted that an imbalance between FPP and GGPP, leading to the disruption of protein prenylation, is one of the key underlying mechanisms causing impaired oocyte quality induced by high cholesterol levels.

## Conclusion

Together, this study uncovers an oocyte-specific role of Ggps1 in the regulation of oocyte maturation and acquisition of developmental competence in mammals as well as environmental challenges to offspring. Therefore, maintaining appropriate cholesterol metabolism and protein prenylation in oocytes benefits female fertility. Supplementation of GGPP might be a potential approach for improving clinic oocyte quality and supporting the growth of healthy babies.

## Experimental procedures

### Animals

C57BL/6J mice, *Zp3-Cre/+* mice, and transgenic *floxed Ggps1* (*Ggps1*^*fl/fl*^) mice were obtained from GemPharmatech (Nanjing). 129S mice were obtained from Charles River (Beijing). Mice with *Ggps1*^*fl/fl*^ were crossed with *Zp3-Cre/+* mice to generate *Zp3-Cre/+; Ggps1*^*fl/fl*^ (hereafter referred to as *Ggps1-ZcKO*) mutants. The mice were genotyped by PCR using the primers shown in Table S1 in the Supporting information. Pregnant female mice at 0.5 to 13.5 days of gestation were sacrificed under isoflurane inhalation followed by cervical dislocation at the indicated time points (0.5 days, n = 6–12; 1.5 days, n = 6–12; 4.5 days, n = 5–6; 12 days, n = 6–8; 13.5 days, n = 4). Pregnant female 129S mice at 3.5 days of gestation were used for embryo transfer experiments (n = 4).

Female C57BL/6J littermates (6-week-old) were fed with normal chow or a high-fat/high-cholesterol diet ad libitum for 10 weeks, 20 weeks, and 24 weeks (n = 5–6/timepoint). At the experimental endpoints, mice were sacrificed, and serum/tissues were harvested. All the mice were housed in ventilated cages on a 12:12 h light-dark cycle at constant temperature (22 °C) and under controlled humidity. Mouse experimental procedures and protocols were approved by the Institutional Animal Care and Use Committee of Nanjing Medical University (Approval number: IACUC2004029) and were conducted in accordance with institutional guidelines for the care and use of laboratory animals.

### Fertility test

A fertility test was carried out by mating 6-week-old female mice of different genotypes with normal adult C57BL/6J males with proven fertility for up to 5 months (n = 5–7). The number of pups for each litter was recorded at birth, and the average cumulative number of pups per female was calculated at the end of the fertility test. For Chow and HF-HCD (HF-HCD for 16 weeks) mice (n = 6–11), female mice were mated with normal adult C57BL/6J males. The number of pups per female was calculated at the first litter.

### Oocyte and embryo collection

Oocyte and embryo collection were carried out in M2 medium (Sigma). To study the ability of oocytes to resume and complete the first meiosis and the kinetics of oocyte meiotic progression, full-grown oocytes (GV oocytes) were isolated from equine chorionic gonadotropin (eCG, Ningbo A Second Hormone Factory)-primed (48 h) 3-week-old female mice and were matured in culture as described previously (51). For mature ovulated oocytes, oocytes were collected from the oviductal ampullae of the mice that were initially primed with eCG for 48 h followed by human chorionic gonadotropin for 13 to 14 h (n ≥ 3/experiment). Early embryos, including zygotes and 2-cell embryos, were collected at day 0.5 poist coitum (dpc) and 1.5 dpc, respectively, from pregnant mice (pregnant mice, n = 5–12/timepoint). Blastocyst implantation was evaluated in 4.5 dpc dams with Evans blue staining (n = 5–6/timepoint).

### Ovarian histological analysis and immunohistochemistry

For histological analysis, ovaries were fixed overnight at 4 °C in 4% paraformaldehyde (Sigma), dehydrated in ethanol (Sinopharm), embedded in paraffin (Sinopharm), and cut into 5 μm sections by paraffin slicing machine (LEICA RM2235). For histological analysis, the sections were subjected to H&E staining according to a standard protocol. Follicles at different stages of development were counted in every third section throughout the whole ovary, and the total number of follicles at each stage was calculated as described previously. To avoid repeated counting of the same follicle, follicles containing an oocyte with visible nucleus were counted (3 weeks, n = 8). The stages of follicles were categorized according to criteria described previously (52).

For IHC, paraffin sections were deparaffinized, rehydrated, and boiled in citrate buffer (pH 6.0) for antigen retrieval. Afterward, paraffin sections were permeabilized, blocked, and incubated with the indicated primary antibodies at 4 °C overnight. Subsequently, the sections were incubated with secondary antibodies for 1 h at room temperature. Ggps1 antibodies were used for IHC (1:100, Thermo Fisher). Images were captured by a microscope (Olympus).

### Immunostaining and confocal microscopic imaging of oocyte staining

Both oocyte and early embryo samples were fixed in 4% paraformaldehyde in PBS for 30 min at room temperature and then permeabilized and blocked for 1 h in PBS containing 0.1% Triton X-100 (Sigma) and 10% fetal bovine serum (FBS) (Thermo Fisher Scientific). The samples were subsequently incubated with primary antibodies (4 °C, overnight) and Alexa Fluor 594/488-conjugated secondary antibodies (room temperature, 30 min) and counterstained with Hoechst 33432 for 20 min. The samples were finally mounted on glass slides and examined under an FV3000 confocal microscope (Olympus). Images for different groups of samples in the same experiment were taken under the same parameters. The primary antibody used for staining oocyte spindles was a monoclonal anti-Tubulin antibody produced in mice. The embryo samples were stained with DAPI (Beyotime).

### Total RNA isolation and Q-PCR

The mouse tissues were carefully isolated, and the samples were frozen quickly and ground to homogeneity in liquid nitrogen. Total cellular RNA was isolated by using TRIzol (Invitrogen) as described in the manufacturer’s instructions and was then subjected to reverse transcription with PrimeScript RT Master Mix (Takara). The steady-state levels of mRNA in ovaries and livers were determined by SYBR Green (Vazyme)-based quantitative real-time PCR analysis using the primer pairs shown in Table S1. The relative changes in the levels of transcripts between the control and experimental groups were analyzed by using the 2^−ΔΔCT^ analysis method, in which 18S rRNA was used as an internal control.

### Western blot analysis

For liver and ovary tissues, total protein was extracted from frozen tissues using RIPA lysis buffer (Tris-HCl 50 mM, NaCl 150 mM, Triton X-100 1% vol/vol, sodium deoxycholate 1% wt/vol, and SDS 0.1% wt/vol pH 7.5) containing a protease/phosphatase inhibitor cocktail (Roche). The protein concentration in each sample was determined using a BCA Protein Assay (Bio-Rad Laboratories). Equal amounts of protein (20 μg) were separated by 8 to 12% SDS-PAGE. For oocyte samples, oocytes were collected according to a standard protocol, lysed in 2× Laemmli sample buffer, and loaded on the same SDS-PAGE gel, and the resolved proteins were transferred to polyvinylidene fluoride membranes (Invitrogen) by standard methods to compare the expression of corresponding proteins between different genotypes or treatments. The immunoblots were blocked with 5% nonfat milk solution and incubated overnight at 4 °C with primary antibodies against Ggps1 (1:400, Thermo Fisher), Rap1A (1:400, Santa Cruz), CYTB (1:1000, Proteintech), ND5 (1:1000, Proteintech), MT-CO1 (1:1000, Proteintech), Tubulin (1:1000, Proteintech), β-Actin (1:1000, Proteintech), LC3 (1:1000, Proteintech), Rab7 (1:500, Santa Cruz), Rac1 (1:400, Santa Cruz), Rhoa (1:400, Santa Cruz), CLPB (1:1000, Abclonal), ABCB7 (1:1000, Abclonal), DAP3 (1:1000, Abclonal, GPD2 (1:1000, Abcam,), and HSP60 (1:1000, Proteintech). Goat anti-rabbit or anti-mouse secondary antibodies (1:5000, Proteintech) were used. Immunodetection was carried out using an enhanced chemiluminescence kit (Tanon).

### Transmission electron microscopy

Mature oocytes isolated from mice that were initially primed with eCG for 48 h followed by human chorionic gonadotropin for 13 to 14 h were fixed in 2.5% glutaraldehyde (Sigma) for 2 h at 4 °C, washed three times in PBS for 15 min each and then embedded in 1.5% agarose. The agarose gel cube (approximately 2 × 2 × 2 mm) containing oocytes was fixed overnight in 2.5% glutaraldehyde and sent to the Biomedical Imaging Core Facility at Nanjing Medical University for transmission electron microscopic analysis (FEI Tecnai G2 Spirit Bio TWIN) using a standard protocol.

### Cell culture, siRNA, and plasmid transfections

Control and *Ggps1-ZcKO* females were mated with fertile C57BL/6J males. Primary MEF cells were isolated from 13.5 dpc from both sexes of embryos using a standard protocol. Each embryo was dissected into 10 ml of sterile PBS and voided of its internal organs, head, and legs. After 30 min of incubation with gentle shaking at 37 °C with 5 ml 0.1% trypsin, the cells were plated in two 100 mm dishes and incubated for 24 to 48 h in Dulbecco's modified Eagle's medium (DMEM) containing 10% FBS and antibiotics under standard conditions. 293T and Aml12 cells were cultured, respectively, in DMEM and DMEM/F12 containing 10% FBS and antibiotics under standard conditions. The siRNA of mouse *Ggps1* and human *GGPS1* were synthesized by Genepharma. Mouse *Dap3-WT* and *Dap3-C363S* were synthesized by GenScript and cloned into the pcDNA3.1-entry vector, which were confirmed by sequencing. Cells were transiently transfected with various expression vectors by using Lipofectamine 2000 reagent (Invitrogen) following the manufacturer’s protocol. After 48 h, cells were harvested and prepared for the next experiments. The empty vector pcDNA3.1 or scramble siRNA was used as a mock transfection control. *Ggps1 siRNA*: CCAGAUUAGAGAUGAUUAUTT (sense). GGPS1 siRNA: CCUGAGCUAGUAGCCUUAGUA (sense).

### Seahorse analysis

MEFs were isolated from WT and *Ggps1-ZcKO* pregnant mice using a standard protocol. Aml12 cells were cultured and transiently transfected with various expression vectors as described above. Oxygen consumption was measured using the XF24 MitoStress Test (Seahorse Bioscience). The OCRs were normalized to the cell numbers using CyQuant (Thermo Fisher), as described previously (53).

### Protein sample preparation, digestion, and tandem mass tag labeling

GV and MII oocytes were isolated from 3-week-old female WT and *Ggps1-ZcKO* mice. GV and MII oocytes from WT and KO mice (three replicates from each condition) were subjected to protein extraction and digestion. In brief, tissues were lysed with protein extraction buffer [8 M urea (Sigma), 75 mM NaCl (Sigma), 50 mM Tris (Sigma), pH 8.2, 1% (vol/vol) EDTA-free protease inhibitor (Roche), 1 mM NaF, 1 mM β-glycerophosphate, 1 mM sodium orthovanadate, 10 mM sodium pyrophosphate (Sigma)] followed by sonication and centrifugation. After cysteine residues reduced with 5 mM DTT (Thermo Fisher) for 25 min at 56 °C, alkylated with 14 mM iodoacetamide (Sigma) for 30 min in the dark and quenched by DTT (Sigma) for 15 min, the proteins were digested overnight at 37 °C with 5 ng/μl trypsin (Promega) and 1 mM CaCl2 (Sigma). The peptides were desalted by tC18 Sep-Pak column (Waters) and labeled by TMT 6-Plex reagents (Thermo Fisher) labeling. After labeling, six GV oocyte samples and six MII oocyte samples were mixed separately and desalted by Waters tC18 Sep-Pak column.

### LC-MS/MS measurements and data processing

Fractionation of TMT-labeled peptide mixture was performed using ACQUITY UPLC M-Class with XBridge BEH C18 column (300 μm × 150 mm, 1.7 μm; 130 Å, Waters). Thirty fractions were collected by using nonadjacent pooling scheme within a 108 min gradient of 3% buffer B (A: 10 mM ammonium formate, pH 10; B: 100% ACN) for 14 min, 3%–8% B for 1 min, 8%–29% B for 71 min, 29%–41% B for 12 min, 41%–100% B for 1 min, 100% B for 8 min, 100%–3% B for 1 min).

For LC-MS/MS analyses, the fractionated peptide samples in 0.1% FA were analyzed using an LTQ Orbitrap Fusion Lumos mass spectrometer (Thermo Fisher) coupled to the Easy-nLC 1200 (Thermo Fisher) with a trap column (75 μm × 2 cm, Acclaim PepMap100 C18 column, 3 μm, 100 Å; DIONEX) and a microcapillary column (75 μm × 25 cm, Acclaim PepMap RSLC C18 column, 2 μm, 100 Å; DIONEX) in an 87 min linear gradient (3%–5% buffer B for 5 s, 5%–15% buffer B for 40 min, 15%–28% buffer B for 34.8 min, 28%–38% buffer B for 12 min, 30% buffer to 100% buffer B for 5 s) using 0.1% FA (buffer A) and 80% ACN, 0.1% FA (buffer B). The Orbitrap Fusion Lumos mass spectrometer was operated in the data-dependent mode. A full survey scan was obtained for the m/z range of 350 to 1500, followed by HCD MS/MS in the resolution of 15,000.

Raw files were searched against the mouse protein sequences obtained from the Universal Protein Resource (UniProt, 2018.07.18) (https://www.uniprot.org/) database by MaxQuant software (version 1.6.5.0) (54). False discovery rate cut-offs were set to 0.01 for proteins and peptides. A protein is considered differentially expressed if *p* value is less than 0.05 by Student’s *t* test, and fold change is greater than 1.2 between groups.

### Subcellular fractionation

The isolation of mitochondria was carried out as previously described (55). The cells cultured in 10 cm dish were washed with PBS and collected in 500 μl fractionation buffer and incubated for 15 min on ice. The cells were then lysed with a 1 ml syringe by passing cell suspension through a 27-gauge needle 30 times (or until all cells are lysed). The initial lysates were on ice for 20 min and centrifuged at 720*g* (3000 rpm) for 5 min. The pellets will contain nuclei, and the supernatants will contain cytoplasm, membrane, and mitochondria. The supernatants were transferred into a fresh tube and centrifuged at 8000 rpm (10,000*g*) for 5 min to pellet the mitochondria. The supernatants were collected as postmitochondrial components (including cytoplasm and membrane fraction).The resulting mitochondrial pellets were then resuspended in the TBS with 0.1% SDS with sonication briefly to homogenize the lysate (3 s on the ice at a power setting of 2-continuous) and subjected to following immunoblotting analysis.

### Triton X-114 extraction of hydrophobic proteins

First, 293T cells transfected with si*Ggps1* and scramble siRNA were subjected to subcellular fractionation to obtain isolated mitochondrial and postmitochondrial components as above. Hydrophobic and hydrophilic proteins were purified using Triton X-114 extraction to determine the membrane localization of target proteins. Then, mitochondria were homogenized in Triton X-114 lysis buffer, and the lysates were centrifuged at 12,000*g* for 15 min at 4 °C. The supernatant was incubated at 37 °C for 5 min until the lysate became turbid and was centrifuged at 12,000*g* for 5 min at room temperature. The upper phase was an aqueous phase containing hydrophilic proteins (water-soluble small G protein), and the lower phase was a detergent phase containing hydrophobic proteins (lipid-soluble small G protein). Using a standard procedure, hydrophobic proteins were precipitated and extracted to the detergent phase by methanol/chloroform precipitation (56). Sample buffer was added, and the mixture was boiled for preparation of the samples for SDS-PAGE and immunoblotting.

### Measurement of total cholesterol and blood glucose

Female C57BL/6J littermates (6-week-old) were fed with normal chow or a high-fat/high-cholesterol diet ad libitum for 10 weeks, 16 weeks, and 24 weeks (n = 5–9/timepoint). Total cholesterol in serum and liver were detected by the total cholesterol assay kit according to the manufacturer’s instructions (Total Cholesterol Colorimetric Assay Kit, Elabscience). In liver, the total cholesterol level was normalized with liver weight. The blood samples of adult male F1 offspring and F1 newborn mice were harvested for blood glucose level assay. The mice blood glucose level of blood samples was measured by New Accu-Chek Active Blood Glucose Monitoring Kit (Roche) manufacturer’s instructions. The sensitivity of total cholesterol and blood glucose assays is 0.29 mmol/l and 0.6 mmol/l, respectively. Both kits are specific to mouse samples. For total cholesterol assay kit, interbatch coefficient of variation ≤8.3%, interbatch coefficient of variation ≤3.1%; for blood glucose assay kit, coefficient of variation ≤3.7%, interbatch coefficient of variation ≤2.0%.

### GTT and ITT

Adult male F1 offspring (3 months, 9 months, n = 6–9) were subjected to GTT and ITT assay. To determine glucose tolerance, mice were injected intraperitoneally with glucose (2 mg/g of body weight) (Sigma) after a 16-h fast. To test insulin sensitivity, mice were given an intraperitoneal injection of insulin (0.7 units/kg of body weight) (Sigma) after a 4-h fast. Blood glucose levels were determined at the indicated times (0, 15, 30, 60, 90, and 120 min) from tail blood using the Accu-Check Glucometer (Roche).

### Statistical analysis

All data are presented as the mean ± SEM and were analyzed using an unpaired two-tailed Student's *t* test between two groups. Differences among multiple groups were tested by one-way ANOVA with Dunnett’s post-hoc test or two-way ANOVA followed by Bonferroni’s post-hoc test. ∗*p* <0.05 was considered to indicate statistical significance. All statistical analyses were performed using Origin 8.0 and GraphPad Prism 5 software.

## Data availability

All unique reagents generated in this study are available from the Lead Contact, C. L. (lichaojun@njmu.edu.cn) with a completed Materials Transfer Agreement. The mouse oocyte RNA-seq data can be found under accession numbers GSE175835. Further information and requests for reagents and resources should be directed to and will be fulfilled by the lead contact.

## Supporting information

This article contains supporting information.

## Conflict of interest

The authors declare that they have no conflict of interest with the contents of this article.
