# Supplementary material for: Oocytes orchestrate protein prenylation for mitochondrial function through selective inactivation of cholesterol biosynthesis in murine species
Source: J Biol Chem. 2023 Aug 21;299(10):105183. doi: 10.1016/j.jbc.2023.105183 (PMC10534227; doi:10.1016/j.jbc.2023.105183)
Supplement: Supporting Figures S1–S7 and Table S1 [file mmc1.pdf]

Supporting Information

Oocytes orchestrate protein prenylation for mitochondrial function through selective inactivation of cholesterol biosynthesis in murine species

Yongjuan Sang, Qiwen Yang, Yueshuai Guo, Xiaofei Liu, Di Shen, Chen Jiang, Xinying Wang, Kang Li, Haiquan Wang, Chaofan Yang, Lijun Ding, Haixiang Sun, Xuejiang Guo*, Chaojun Li**

These authors contributed equally: Yongjuan Sang, Qiwen Yang

Supplemental Figures and Figure Legends

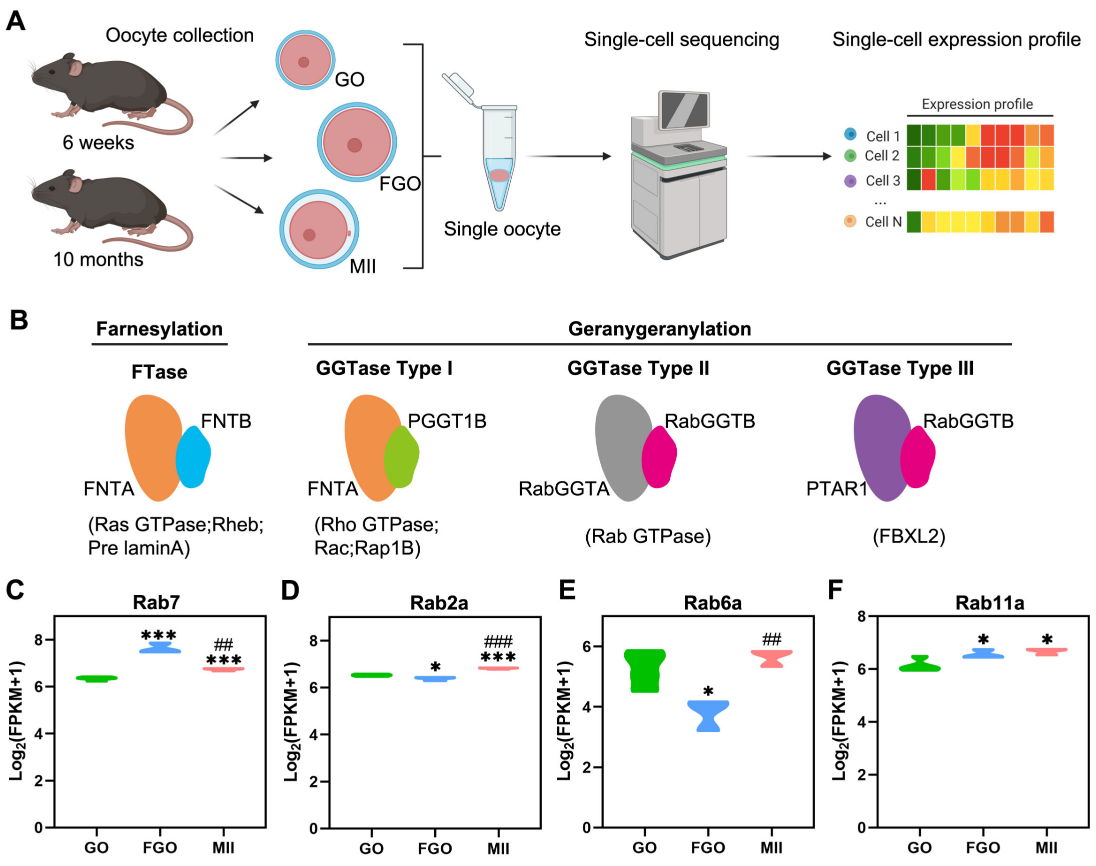

Figure S1. Small GTP proteins undergoing prenylation showed dynamic expression patterns among oocytes maturation. Related to Figure 1.

(A) Schematic illustration showing the preparation of oocytes (GO, FGO, and MIIO) from 6-week-old mice for RNA-seq (n = 3-4).

(B) Schematic overview of prenyltransferases and their substrates involved in protein prenylation. Prenyltransferases are composed of α and β subunits. Four classes of human prenyltransferases with α (PTAR1, FNTA, and RabGGTA) and β (FNTB, PGGT1B, and RabGGTB) subunits have their substrates because of different recognition sequences, and accessory proteins. GGTase type III is a novel type prenyltransferase, identified recently.

(C-F) Relative levels of genes encoding small GTP proteins in mouse oocytes at different stages (3 weeks, n = 3-4, $*p < 0.05$, $***p < 0.001$, $^{\#}p < 0.05$, $^{\#\#}p < 0.01$, $^{\#\#\#}p <$

0.001). *$p < 0.05$, ***$p < 0.001$, compared with the GO by Student's t test. $^{\#}p < 0.05$, $^{\#\#}p < 0.01$, $^{\#\#\#}p < 0.001$ compared with the FGO by Student's t test. The data represent the mean ± SEM.

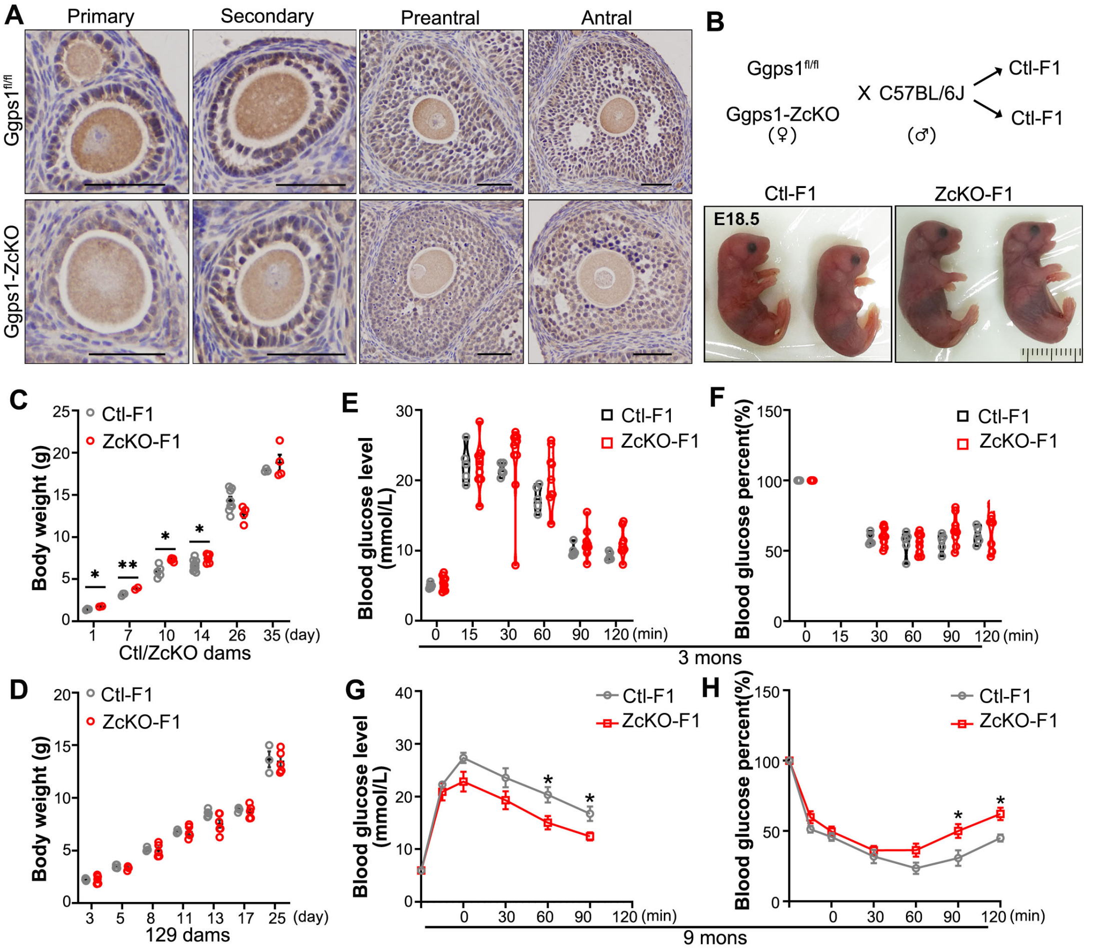

Figure S2. ***Ggps1*** **deficiency in growing oocytes impairs female fertility and results in offspring metabolic defects in mice. Related to Figure 2.**

(A) IHC of Ggps1 in 23-d-old *Ggps1$^{fl/fl}$* and *Ggps1-ZcKO* ovaries. Staining of Ggps1 in oocytes at different stages is presented (n = 4). Scale bars, 100 μm.

(B) Representative images showing conceptuses (Ctl-F1 and ZcKO-F1) at E18.5 obtained from *Ggps1$^{fl/fl}$* and *Ggps1-ZcKO* dams. The females were mated with C57BL/6J male mice.

(C-D) Postnatal body weights of Ctl-F1 and ZcKO-F1 before 5 weeks from *Ggps1$^{fl/fl}$*

and *Ggps1-ZcKO* females (n = 3-5, *$p < 0.05$, **$p < 0.01$). F1 in (C) was produced by natural parturition, while F1 in (D) was produced by embryo transfer, for which 129 females were surrogate mothers.

(E-F) Blood glucose level and percentages during the GTT and ITT in the Ctl-F1 and ZcKO-F1 groups (the tested F1 was produced by embryo transfer) at 3 months (n = 5-9, *$p < 0.05$).

(G-H) Blood glucose level and percentages during the GTT and ITT in the Ctl-F1 and ZcKO-F1 groups (the tested F1 was produced by embryo transfer) at 9 months (n = 5-9). *$p < 0.05$, **$p < 0.01$, compared with the WT or control by Student's t test. The data represent the mean ± SEM.

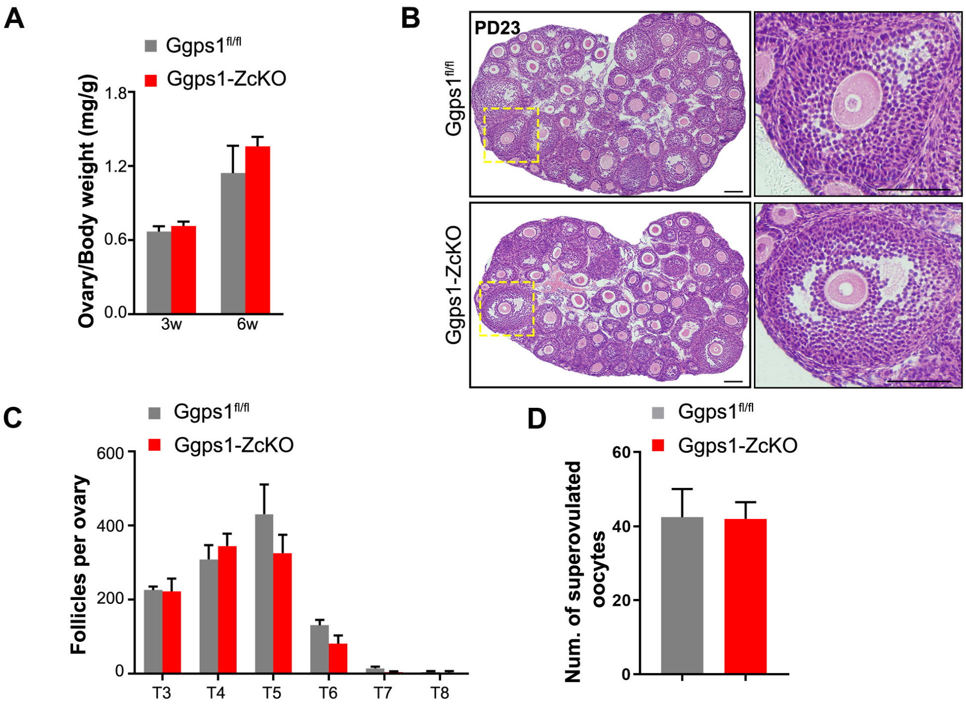

Figure S3. Follicle development is not affected by *Ggps1* deficiency in growing oocytes

(A) Ratios of ovary weight to body weight in *Ggps1*$^{fl/fl}$ and *Ggps1-ZcKO* mice at 3 weeks (n = 3-4, *$p < 0.05$).

(B) Representative images showing ovarian morphology by HE staining in *Ggps1*$^{fl/fl}$ and *Ggps1-ZcKO* mice at PD23 (n = 8). Scale bar, 100 μm.

(C) Quantification of follicles at different stages in *Ggps1*$^{fl/fl}$ and *Ggps1-ZcKO* ovaries at 3 weeks (n = 8, *$p < 0.05$).

(D) Numbers of superovulated oocytes in 3-week-old *Ggps1*$^{fl/fl}$ and *Ggps1-ZcKO* mice treated with PMSG and hCG (n = 7-10, *$p < 0.05$). *$p < 0.05$, compared with the WT or control by Student's t test. The data represent the mean ± SEM.

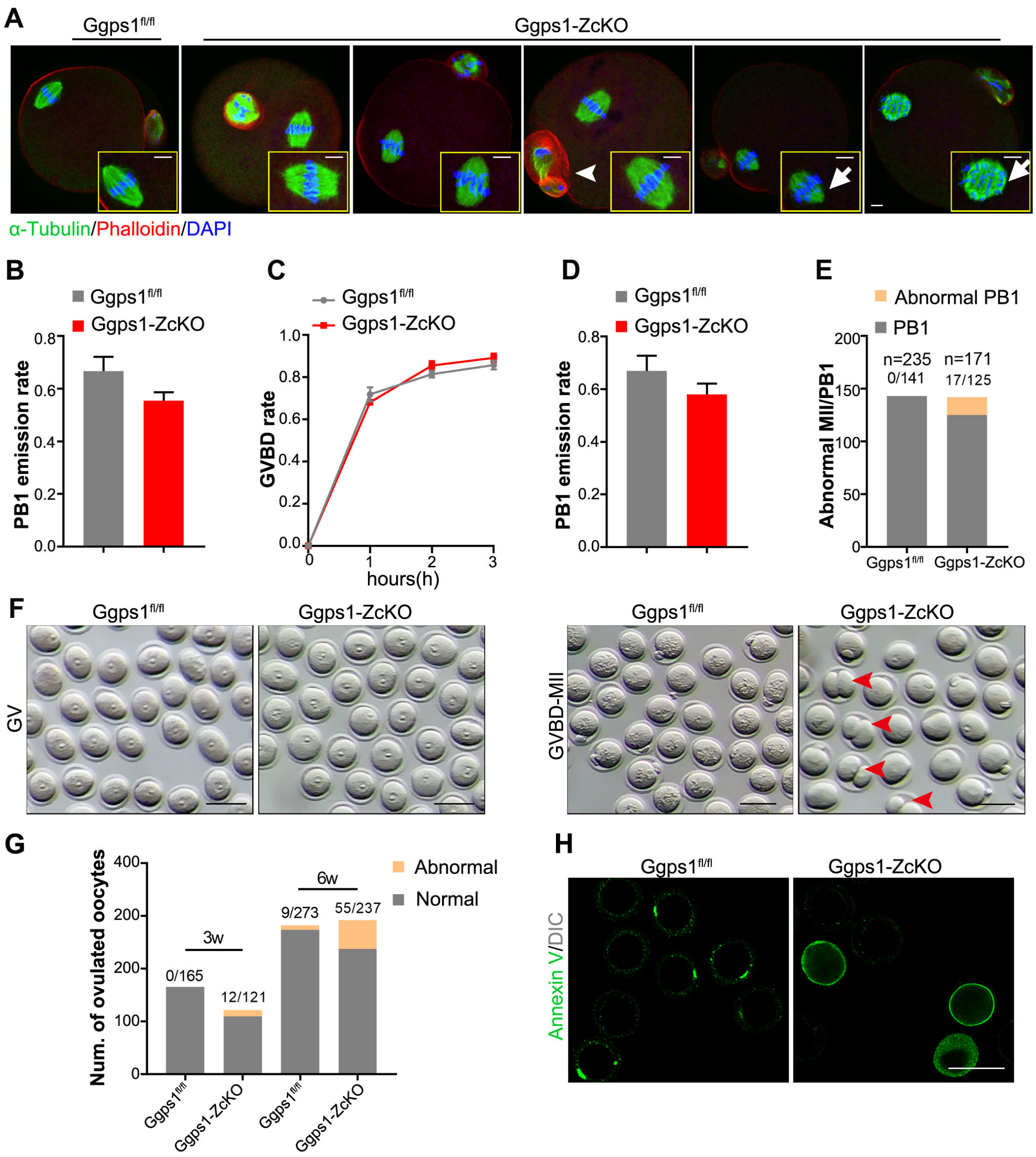

Figure S4. *Ggps1* deficiency in growing oocytes results in oocyte meiosis defects

(A) Micrographs showing typical meiotic stages prevalent in *Ggps1$^{fl/fl}$* and *Ggps1-ZcKO* ovulated oocytes. Normal MII stage in *Ggps1$^{fl/fl}$* mice. Abnormal MII stage with a deformed spindle and misaligned chromosomes in *Ggps1-ZcKO* mice (3 weeks, n = 3-5, 100 oocytes/group). Scale bar, 10 μm. Tubulin was used to stain spindle and phalloidin was used to stain F-actin.

(B) First polar body (PB1) extrusion rate in *Ggps1$^{fl/fl}$* and *Ggps1-ZcKO* ovulated oocytes (3 weeks, n = 3-5, 100 oocytes/group, *$p < 0.05$).

(C-E) Kinetics of PB1 extrusion in oocytes after release from meiotic arrest and maturation in culture and quantification of the proportion of oocytes shown in (C) reaching normal MII. First polar body (PB1) extrusion and abnormal MII formation in oocytes that underwent maturation *in vitro* were detected (3 weeks, n = 3-5, 150-300 oocytes/group, $*p < 0.05$).

(F) Representative images showing GV and MII oocytes *in vitro* maturation. GV oocytes were collected from 3-week-old mice (n = 3-5, 150 -300 oocytes/group). Scale bar, 100 μm.

(G) Quantification of ovulated oocytes at 3 weeks and 6 weeks in *Ggps1*$^{fl/fl}$ and *Ggps1-ZcKO* mice, respectively (3 weeks, n = 3-5, 121-165 oocytes/group; 6 weeks, n = 6-8, 237-273 oocytes/group).

(H) Representative micrographs of Annexin V staining in oocytes ovulated from 3-week-old *Ggps1*$^{fl/fl}$ and *Ggps1-ZcKO* mice (3 weeks, n = 3-5, 100 oocytes/group,). Scale bar, 100 μm. $*p < 0.05$, compared with the WT or control by Student's t test. The data represent the mean ± SEM.

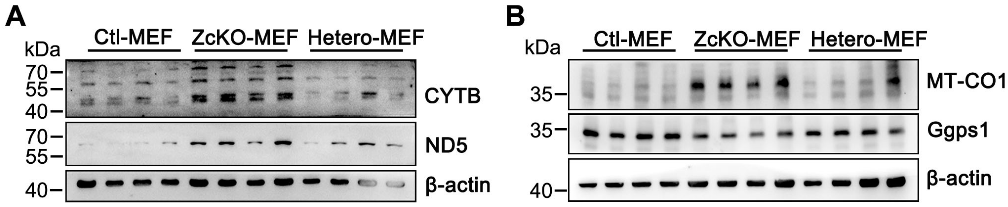

Figure S5. *Ggps1* is essential for mitochondrial quality control in growing oocytes and embryo development. Related to Figure 3.

(A-B) Western blot analysis of mitochondrial respiratory chain subunits in MEFs from embryos at E13.5 from *Ggps1$^{fl/fl}$* and *Ggps1-ZcKO* pregnant mice (pregnant mice, n = 3).

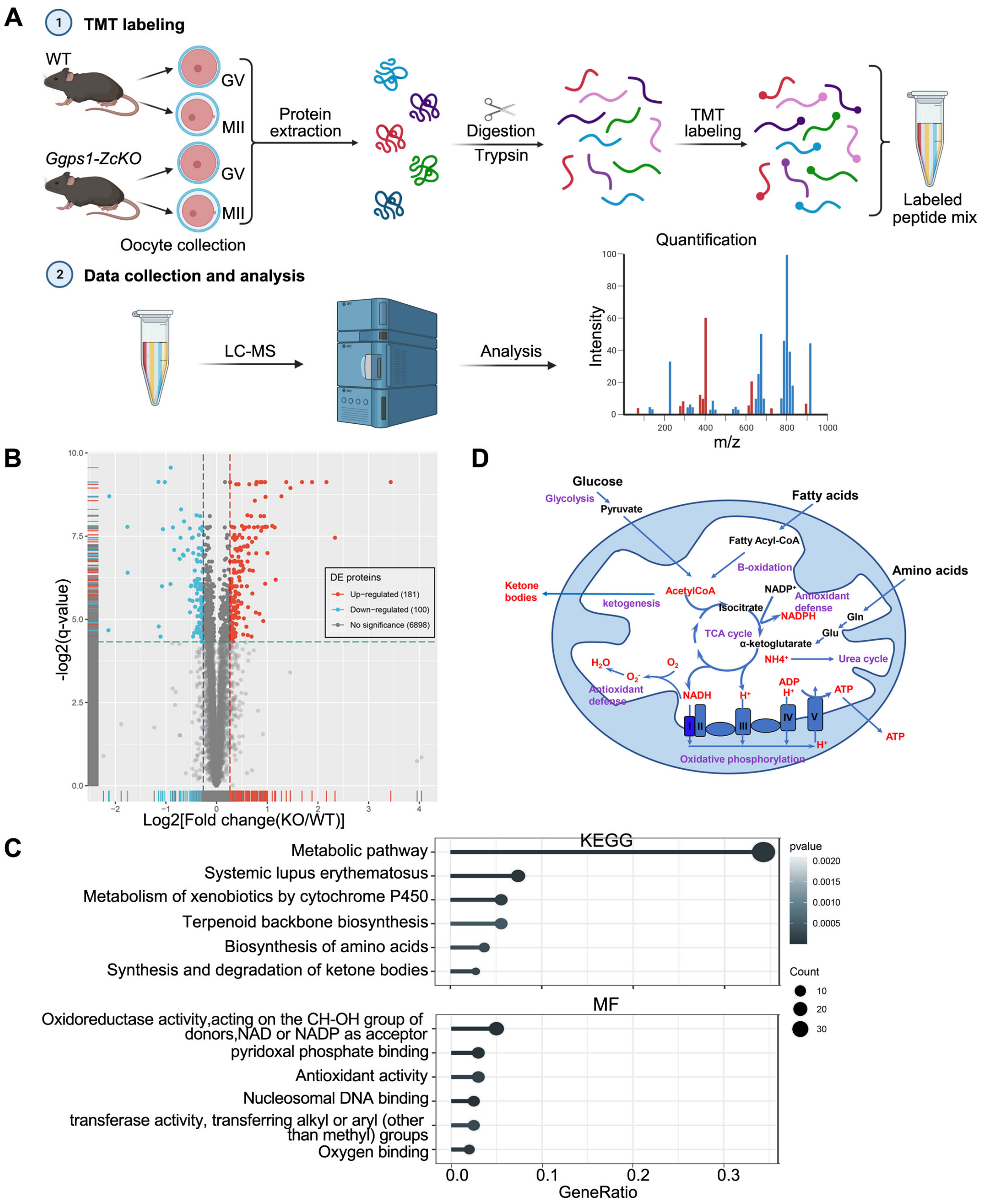

Figure S6. Proteomics reveals more alterations of proteins in the MII stage than in the GV stage. Related to Figure 4.

(A) Experimental workflow used in the proteomics experiment. GV and MII oocytes were collected from WT and Ggps1-ZcKO mice. Scheme was created with ©BioRender.com.

(B) Distributions of proteins with significant expression level differences of various magnitudes between *Ggps1$^{fl/fl}$* and *Ggps1-ZcKO* GV oocytes as detected by LC-MS/MS.

The number of changed proteins in each category of fold change is indicated above each bar (3 weeks, n = 60-80, 2000 oocytes per group, fold change ⩾ 1.2).

(C) Heatmaps illustrating the enriched terms (KEGG terms and MF) identified by LC-MS/MS in *Ggps1*$^{fl/fl}$ and *Ggps1-ZcKO* MII oocytes (*p < 0.05, fold change ≥ 1.2).

(D) Schematic images showing the relationships between different metabolites (glucose, fatty acids, amino acids) and mitochondria. *p < 0.05, compared with the WT or control by Student's t test.

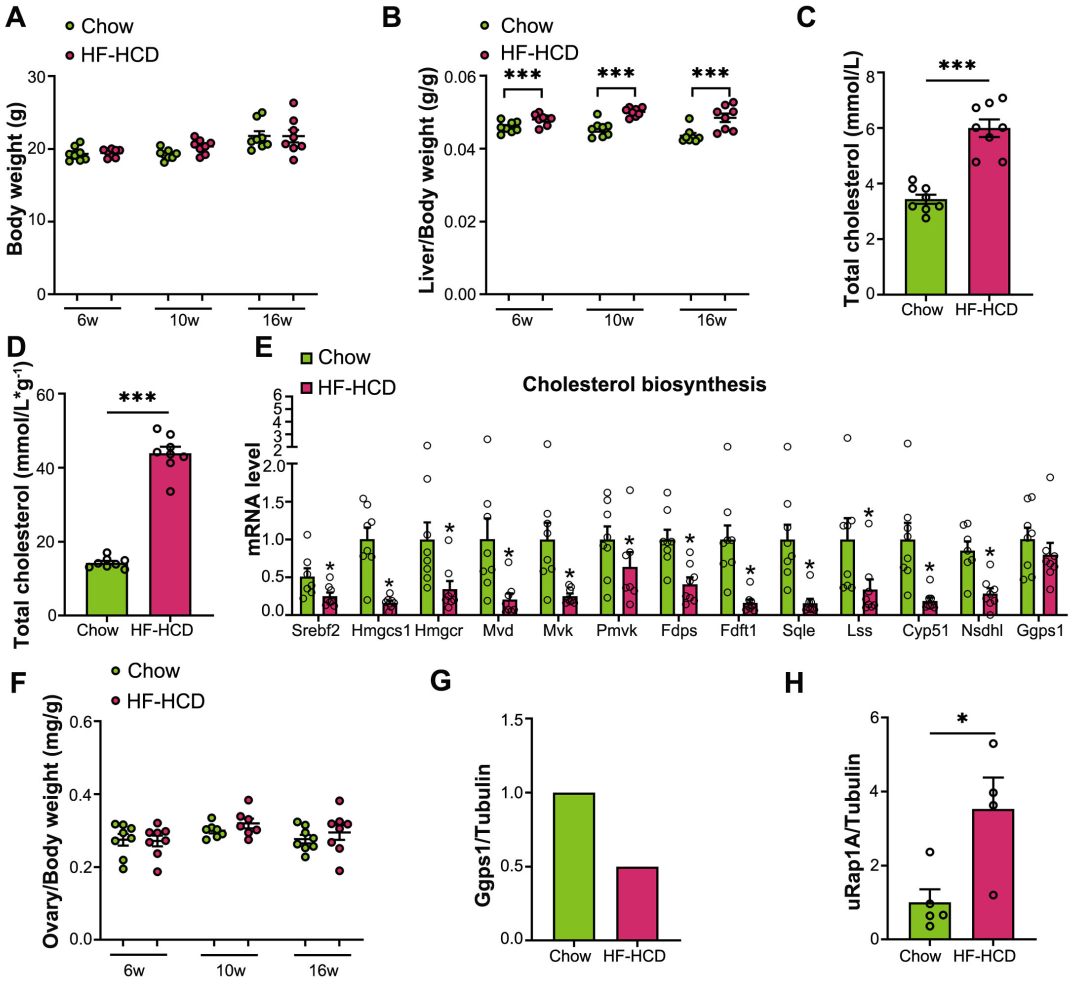

Figure S7. Hypercholesterolemia disrupts ovarian protein prenylation balance and results in offspring metabolic dysfunction. Related to Figure 6.

(A) Body weights of the females fed normal chow or a HF-HCD for 6 weeks, 10 weeks, or 16 weeks (n = 8, **p* < 0.05).

(B) Ratios of liver weight to body weight in mice fed normal chow or a HF-HCD for 6 weeks, 10 weeks, or 16 weeks (n = 8, ****p* < 0.001).

(C-D) Total cholesterol levels in the blood and liver after HF-HCD feeding for 10 weeks (n = 8, ****p* < 0.001).

(E) mRNA levels of cholesterol biosynthesis-related genes in the livers of mice fed normal chow or a HF-HCD for 10 weeks (n = 8, **p* < 0.05).

(F) Ratios of ovary weight to body weight for mice fed normal chow or a HF-HCD for

10 weeks, 10 weeks, or 16 weeks (n = 8, *$p < 0.05$).

(G) Relative protein expression of Ggps1 in the oocytes of mice fed normal chow or a HF-HCD for 16 weeks (~120 oocytes from 5-7 mice in each group).

(H) Prenylation levels of the Rap 1A protein in mice fed normal chow or a HF-HCD for 10 weeks (n = 5, *$p < 0.05$). *$p < 0.05$, **$p < 0.01$, ***$p < 0.001$, compared with the WT or control by Student's t test. The data represent the mean ± SEM.

Table S1. List of primer sequences used for real-time PCR analysis and genotyping.

Primers	Sequences (5'to3')
Srebf2-F	GCAGCAACGGGACCATTCT
Srebf2-R	CCCCATGACTAAGTCCTTCAACT
Hmgcr-F	AGCTTGCCCGAATTGTATGTG
Hmgcr-R	TCTGTTGTGAACCATGTGACTTC
Mvd-F	ATGGCCTCAGAAAAGCCTCAG
Mvd-R	TGGTCGTTTTTAGCTGGTCCT
Fdps-F	GGAGGTCCTAGAGTACAATGCC
Fdps-R	AAGCCTGGAGCAGTTCTACAC
Fdft1-F	ATGGAGTTCGTCAAGTGTCTAGG
Fdft1-R	CGTGCCGTATGTCCCCATC
Sqle-F	ATAAGAAATGCGGGGATGTCAC
Sqle-R	ATATCCGAGAAGGCAGCGAAC
lss-F	TCGTGGGGGACCCTATAAAAC
lss-R	CGTCCTCCGCTTGATAATAAGTC
Cyp51-F	GACAGGAGGCAACTTGCTTTC
Cyp51-R	GTGGACTTTTCGCTCCAGC
Nsdhl-F	TCATGGTGAATCAAAGCGAGG
Nsdhl-R	CCGGGGGTTATCAAAGCCTTG
Ggps1-F	TTCACCAACACCTGTAACTC
Ggps1-R	TTATTGACAAGCCCAGAGC
Cre-F	TGCCACGACCAAGTGACAGCAATG
Cre-R	AGAGACGGAAATCCATCGCTCG
Ggps1-loxp-F	AATTGTGTGTGGTAGGGGTA
Ggps1-loxp-R	AACTTGCTTCAGAACTGAGC
